# Selective Hydrogenation of the Carbonyls in Furfural and 5-Hydroxymethylfurfural Catalyzed by PtNi Alloy Supported on SBA-15 in Aqueous Solution Under Mild Conditions

**DOI:** 10.3389/fchem.2021.759512

**Published:** 2021-09-29

**Authors:** Ge Gao, Zhicheng Jiang, Changwei Hu

**Affiliations:** ^1^ College of Biomass Science and Engineering, Sichuan University, Chengdu, China; ^2^ National Engineering Research Center of Clean Technology in Leather Industry, Sichuan University, Chengdu, China; ^3^ Key Laboratory of Green Chemistry and Technology, Ministry of Education, College of Chemistry, Sichuan University, Chengdu, China

**Keywords:** PtNi alloy, SBA-15, selective hydrogenation, furfural, 5-hydroxymethylfurfural

## Abstract

Valuable furfuryl alcohol (FFA) and 2,5-dihydroxymethylfuran (DHMF) could be produced by selective hydrogenation of biomass-derived furfural (FF) and 5-hydroxymethylfurfural (HMF) with high atom economy. In this study, SBA-15 (a kind of mesoporous silica molecular sieve)-supported low metal loading (3 wt% total metal content) PtNi alloy catalyst (PtNi/SBA-15) was synthesized via two steps, including the generation of PtNi alloy by hydrothermal method, and the immobilization of PtNi alloy on SBA-15. PtNi/SBA-15 has ordered mesoporous structure with high surface area, and high dispersion of the PtNi alloy with the formation of Pt^δ−^-Ni^δ+^ surface pairs on SBA-15, which benefit hydrogen activation and selective carbonyl hydrogenation. The selective hydrogenation of FF and HMF over PtNi/SBA-15 in water solvent at 303 K with 1.5 MPa H_2_ within 2 h, could respectively yield 64.6% FFA with 77.0% selectivity, and 68.2% DHMF with 81.9% selectivity. Besides, PtNi/SBA-15 exhibited a satisfactory water resistance and stability after recycling at least five runs.

## 1 Introduction

Preparation of value-added chemicals from renewable lignocellulosic biomass has aroused wide public concerns, since it can mitigate the over-reliance on fossil resources and the related environmental issues (Fukuoka and Dhepe, 2006; Jiang et al., 2018; Remón et al., 2018; Han et al., 2019). Furfural and 5-hydroxymethylfurfural are the most important platform chemicals which could be derived from hemicellulose and cellulose *via* hydrolysis and subsequent dehydration (Mika et al., 2018; Hui et al., 2019; Luo et al., 2019; Chen et al., 2021; Shi et al., 2021). Due to their diverse double bonds, that is, C=O and C=C, FF and HMF could be further converted into different valuable chemicals (Sharma et al., 2013; Fulajtárova et al., 2015; Sun et al., 2019; Herrera et al., 2020; Zhu et al., 2020). Especially, because of the wide applications of furfuryl alcohol (FFA) and 2,5-dihydroxymethylfuran (DHMF) on resins and farm chemicals (Gandini, 1990; Gandini, 2010), the selective conversion of FF/HMF to FFA/DHMF has received much interests. Traditionally, FFA and DHMF could be obtained from FF and HMF by Cannizzaro disproportionation reaction without H_2_ (Hazlet and Callison, 1944; Chacón-Huete et al., 2018). However, Cannizzaro reaction needs strong base (e.g., NaOH, KOH) as reaction medium and the molar quantity of by-products is always equal to that of the target products, which would cause environmental pollution, separation trouble, low selectivity and low atom economy. The selective C=O hydrogenation of FF/HMF to produce FFA/DHMF provides one atom economic way, which has been widely researched to replace the Cannizzaro reaction (Supplementary Table S1). However, the hydrogenation of the carbonyls is always accompanied with the further C=C hydrogenation of furan rings into tetrahydrofuran rings (Lima et al., 2017; Tan et al., 2019; Wu et al., 2019). For example, 58.0% yield of FFA could be obtained from the hydrogenation of FF over Ni/C catalyst, while 21.0% yield of tetrahydrofurfuryl alcohol (THFA) was generated in the meantime (Wu et al., 2019). Moreover, the hydrogenolysis of hydroxyl and furan ring groups often occurs simultaneausly with C=O hydrogenation to form other by-products, such as 2-methyltetrahydrofuran (2-MTHF), 2-methylfurfuran (2-MF), 2-pentanol, 2,5-dimethylfuran (DMF), and 2,5-dimethyltetrahydrofuran (DMTHF), attenuating the selectivity to FFA/DHMF (Perez and Fraga, 2014; Mitra et al., 2015; Srivastava et al., 2015; Chacón-Huete et al., 2018; Tan et al., 2019; Yang et al., 2019). It is desirable but highly challenging to selectively catalyze the hydrogenation of carbonyls while keeping the furan rings and hydroxyl unaffected.

Among the reported catalytic systems for FF/HMF hydrogenation and hydrogenolysis, monometallic catalysts (e.g., Pt, Pd, Ni, Cu) were widely employed and investigated (Sitthisa and Resasco, 2011; Chen et al., 2016; Guo et al., 2016; Lima et al., 2017; Fan et al., 2020; Yang et al., 2020). For example, Fan et al. (2020) reported that FF hydrogenation over 10 wt% Pt/C catalyst could yield 14% FFA with 71.2% selectivity at 413 K. Sitthisa and Resasco (2011) reported that 69% conversion of FF and 68% selectivity to FFA could be achieved on 10 wt% Cu/SiO_2_ at 503 K . However, monometallic catalyst with relatively high metal loading always accompanied with low metal dispersion, consequently resulting in the slow reaction rate, high reaction temperature and poor selectivity to FFA/DHMF. In order to improve the catalytic performance of monometallic catalysts, the bimetallic nanoalloys have been widely studied and attracted more attention in the last decades (Fang et al., 2018; Xie et al., 2020; Xiao et al., 2021). The bimetallic nanoalloys are significantly different from the two bulk metals and take on intrinsic features, including tunable components and ratios, variable constructions, reconfigurable electronic structures, and optimizable performances. In addition, the bimetallic synergy effect in nanoalloy adjusts the spatial structure and electronic state around the metal active center, which could make the dispersion of active sites higher and further improve the catalytic hydrogenation ability to obtain the target product (Kijenski and Winiarek, 2000; Wu et al., 2012; Jiang et al., 2014; Fang et al., 2018). Among the various bimetallic nanoalloys, PtNi nanoalloys with high activity and stable properties were widely used to catalyze C=O hydrogenation reactions (Wu et al., 2012; Jiang et al., 2014). For example, Jiang et al. reported that PtNi alloys enhanced the selective C=O hydrogenation of cinnamaldehyde in alcohol, and the hydrogenation of C=C double bond of the α,β-unsaturated aldehydes was suppressed (Jiang et al., 2014). Wu et al. (2021) reported that PtNi hollow nanoframes could catalyze the FF hydrogenation with 99% yield of FFA at 373 K in isopropanol solution. However, it is always hard to separate or recycle these expensive and nano-size PtNi catalysts. The nanoparticles with smaller size also have high surface energy, and are easy to aggregate between inter-particles, leading to poor stability and low utilization efficiency (Chen et al., 2016). In addition, organic solvents were used as the reaction medium in these efficient PtNi nanoalloys catalytic systems, which potentially cause harm to both human bodies and environment. It is still a tough task to develop a water-resistant catalyst due to the various side reactions such as rearrangement during the aqueous-phase hydrogenation (Wu et al., 2019). Compared to harsh conditions, mild conditions could improve the selectivity to C=O hydrogenation and suppress the other side reactions (Chen et al., 2016; Chen et al., 2018). It was found (Wu et al., 2019) that Pt(3)Ni(1)/C and Pt(3)Ni(3)/C could catalyze the selective C=O hydrogenation of FF to FFA with 80% selectivity and the FF hydrogenation to THFA with 93% selectivity in water at 308 K within 12 h, respectively. It is an effective and economic method using active carbon as support which makes metal dosage obviously decreased, while keeping high activity of hydrogenation in aqueous phase. Although the Pt(x)Ni(y)/C catalysts achieved a great progress in the FF hydrogenation under mild conditions without organic solvents, the selective C=O hydrogenation of HMF and the alloying degree of PtNi nanoalloys are still required to be studied. Furthermore, active carbon as support has some disadvantages (e.g., normal mechanical strength, low heat resistance, and difficult removal of deposited carbon), limiting its reuse and industry application.

**FIGURE 1:**
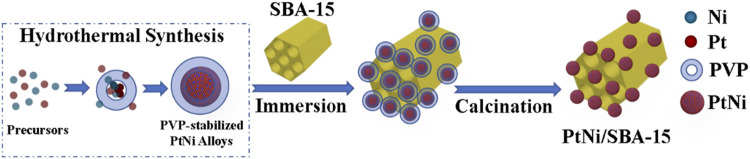
Schematic diagram for the preparation of the PtNi/SBA-15 catalyst.

Given this background, we design a SBA-15 (Santa Barbara Amorphous-15, a stable mesoporous silica molecular sieve, which was developed by researchers at the University of California at Santa Barbara) supported PtNi nanoalloys catalyst for the C=O hydrogenation of FF/HMF in water under mild conditions, since SBA-15 with advantageous feature (e.g., high heat resistance, variable framework compositions, and low diffusion barrier for the reactants) has been reported to efficiently adsorb carbonyl groups, which could further promote the selective carbonyl hydrogenation (Zhao et al., 1998; Huang et al., 2014; Deng et al., 2019; Samikannu et al., 2020). The catalyst was synthesized by a two-step method without the use of gaseous hydrogen or NaBH_4_ as reductant (Figure 1). The structural features of PtNi/SBA-15 were analyzed by BET, XRD, XPS, TEM, etc. Then, the as prepared catalyst was applied for the hydrogenation of the carbonyls in FF/HMF to obtain FFA/DHMF. In addition, Pt/SBA-15, Ni/SBA-15 and Pt-Ni/SBA-15 were prepared by incipient impregnation method, and the catalytic hydrogenation efficiencies were compared to study the influence of PtNi alloys on the activity of C=O hydrogenation. Finally, the recycle use of PtNi/SBA-15 was conducted for five times to examine its stability. The advantages of the PtNi/SBA-15 catalytic system, such as using water as green reaction medium without organic solvents, lower reaction temperature and lower H_2_ pressure, would promote its industry application.

## 2 Experimental Section

### 2.1 Materials

Polyvinylpyrrolidone (PVP, Mw = 8,000), chloroplatinic acid hydrate (H_2_PtCl_6_·6H_2_O, 99%) and furfural (FF, 98%) were purchased from Shanghai Macklin Biochemical Co., Ltd. 5-hydroxymethylfurfural (HMF, 95%) was purchased from Tokyo Chemical Industry Co., Ltd. Nickel(Ⅱ) chloride hexahydrate (NiCl_2_·6H_2_O, 99%) and glycine (99%) were purchased from Adamas-Beta. SBA-15 powder was purchased from Chengdu Sinovida Biopharmaceutical Co., Ltd. Ultrapure water (18.25 MΩ cm^−1^, 298 K) was obtained from Labpure water system (Aike, China). All chemicals were used as received without further purification.

### 2.2 Catalysts Preparation

3 wt% PtNi/SBA-15 catalyst was prepared via a two-step synthesis method presented in Figure 1. In the first step, 220 mg PVP and 18 mg glycine were dissolved in 2 ml ultrapure water, and then mixed with 0.5 ml H_2_PtCl_6_ solution (0.1 M) and 0.5 ml NiCl_2_ solution (0.1 M). After stirring for 5 min at room temperature, the resulting homogeneous yellow solution was transferred to a 35 ml thick-walled glass tube. The sealed vessel was heated at 453 K for 90 min, and then cooled down to room temperature. The PtNi preparation process was shown in Supplementary Figure S5. The products were washed out with ethanol/water (1:1, v/v) and a PtNi alloy colloidal solution was obtained. In the second step, 330 mg SBA-15 powder was immersed in the as prepared colloidal solution, ultrasonically dispersed for 30 min and stirred for 4 h at room temperature. After impregnation for 8 h, the resulting sample was desiccated in an oven at 353 K for 8 h. The XRD of PtNi alloys and TGA results of PtNi/SBA-15 were displayed in Supplementary Figures S6, S7, respectively. Finally, the sample was grinded into powder (<100 mesh) and calcined in air at 673 K for 1 h (heating rate at 2 K min^−1^) to obtain PtNi/SBA-15 catalyst, which was used directly without further reduction.

In comparison, 3 wt% Pt-Ni/SBA-15, 3 wt% Pt/SBA-15, and 3 wt% Ni/SBA-15 were prepared by incipient impregnation method, where SBA-15 powder was immersed into the relevant H_2_PtCl_6_-NiCl_2_ or H_2_PtCl_6_ or NiCl_2_ solutions, respectively. The H_2_PtCl_6_-NiCl_2_ solution was mixed from H_2_PtCl_6_ and NiCl_2_ solutions (the molar ratio of Pt/Ni = 1). After 8 h of impregnation, the resulting samples were desiccated in an oven at 353 K for 8 h. Then the samples were grinded into powder and calcined in air at 673 K for 1 h (heating rate at 2 K min^−1^). Prior to the experiments, these catalysts were reduced at 673 K for 1 h under H_2_ with a flow rate of 30 ml min^−1^.

### 2.3 Catalyst Characterization

The textural properties of catalysts were determined by N_2_ physisorption measurements (Micromeritics ASAP 2460 analyzer). The specific surface areas, average pore diameter and pore volumes were obtained by BET (Brunauer-Emmett-Teller) equation and BJH (Barrett-Joyner-Halenda) method. Prior to the analytical test, 0.1 g sample was firstly heated under vacuum at 393 K for 2 h and further evacuated at 573 K for 2 h to remove the adsorbed impurities. The metal loading of the catalysts was analyzed by ICP-AES (Germany Kleve Spectro). Prior to measurement, the catalysts were pretreated with aqua-regia at 423 K for 4 h to dissolve the Pt and Ni species. XRD measurements of the catalysts were performed using an XRD-6100 (SHIMADZU) instrument with monochromatic CuKα radiation over the scanning range (2θ) of 0.5°∼5° and 15°∼85° (10 min^−1^). Thermogravimetric analysis (TGA) was performed using NETZSCH STA 449 F5 System (Germany) from 323 to 1,023 K in air atmosphere (heating rate: 10 K min^−1^). TEM images of the catalysts were collected from a Tecnai G2F20 (FEI, German). HRTEM and HAADF-STEM images of the catalysts were acquired from a Talos F200X (FEI, German) equipment. STEM-EDX Mapping analysis of the samples were conducted on the Talos F200X equipment equipped with Super X detector. Before analysis, the samples were ultrasonically dispersed in ethanol/water for 15 min at room temperature, and then deposited onto the copper grids for analysis. XPS measurements of the catalysts were carried out using an Axis Ultra DLD (KRATOS) spectrometer with Al-Kα X-ray radiation. The C 1s peak was set to 284.6 eV to internally calibrate the energy scale and the linear background was subtracted from all spectra.

### 2.4 Catalytic Hydrogenation

For a typical catalytic activity test, 1.25 mmol substrate, 50 mg PtNi/SBA-15, and 20 ml H_2_O were successively added to a 50 ml autoclave reactor equipped with a stirrer. The autoclave was then sealed and pressurized to 1.5 MPa with H_2_ after three times of H_2_ replacement. Then the reactor was heated to the designated temperature (303 K). When the reactor achieved the designated temperature, the internal pressure was 1.5 MPa. After a designed holding time (2 h), the autoclave was fetched out and cooled down to room temperature. The reaction mixture was poured out and the solid catalyst and the liquid were separated by filtration. All the results were summarized in Supplementary Tables S4–S7.

### 2.5 Analysis of the Products

ESI-MS was used to qualitatively determine the liquid products. The instrument (LCMS-IT-TOF, Shimadzu) was operated in continuum mode with the configured parameters (4.5 kV of ionization voltage, 533 K of interface temperature, 1.5 L min^−1^ of nebulizer gas (N_2_) flow, and 1.6 kV of detector voltage.)

The liquid products were quantitatively analyzed by HPLC (Agilent 1260 Infinity), equipped with a variable wavelength detector, a refractive index detector and an aminex column (Model HPX-87 P, 300 mm × 7.8 mm, Bio-Rad). The fluent was ultrapure water at a flow rate of 0.6 ml min^−1^, and the column temperature was maintained at 353 K. The concentrations of all components in the reaction liquid were determined based on the standard calibration curves with about 3% relative percent deviation (RPD). The conversion of the reactant (C), yield (Y), and selectivity (S) of products are defined as follows:

$$\begin{array}{c} C\;\left(\%\right)=\frac{n_{\mathrm{initial}}\;-\;n_{\mathrm{final}}}{n_{\mathrm{initial}}}\times 100 \end{array}$$ (1)

$$\begin{array}{c} Y\;\left(\%\right)=\frac{n_{\mathrm{product}}}{n_{\mathrm{initial}}}\times 100 \end{array}$$ (2)

$$\begin{array}{c} S\;\left(\%\right)=\frac{n_{\mathrm{product}}}{n_{\mathrm{initial}}-\;n_{\mathrm{final}}}\times 100 \end{array}$$ (3)

where ***n***_***initial***_ represents the moles of initial reactants (FF/HMF) before reaction, ***n***_***final***_ represents the moles of reactants (FF/HMF) after reaction, and ***n***_***product***_ represents the moles of each product after reaction.

The turnover number (TON) and turnover frequency (TOF) were calculated by the following equations:

$$\begin{array}{c} metal\;dispersion\;\left(\%\right)=\frac{6.59\;\times \;\mathrm{site}\;\mathrm{density}}{\mathrm{average}\;\mathrm{diameter}\;\mathrm{of}\;\mathrm{metal}} \end{array}$$ (4)

$$\begin{array}{c} TON=\frac{n_{\mathrm{initial}}\;-\;n_{\mathrm{final}}}{\mathrm{metal}\;\mathrm{dispersion}\;\times \;n_{\mathrm{metal}}} \end{array}$$ (5)

$$\begin{array}{c} TOF\left(h^{-1}\right)=\frac{n_{\mathrm{initial}}-n_{\mathrm{final}}}{\mathrm{metal}\;\mathrm{dispersion}\;\times n_{\mathrm{metal}}\times t} \end{array}$$ (6)

In Eq. 4, the metal dispersion for bimetallic catalyst was calculated based on TEM analysis according to the literatures, where the value 6.59 was obtained from calculation by assuming spherical metal crystallites of uniform diameter (Jin et al., 2008; Chang et al., 2020; Zhou et al., 2020). The site density was calculated from the average diameter of platinum and nickel crystallites estimated by TEM (Wu et al., 2012). In Eqs 5, 6, ***n***_***initial***_ and ***n***_***final***_, respectively represents the moles of reactants (FF/HMF) before and after reaction, the reaction time (t) used here was 10 min, and ***n***_***metal***_ represents the total moles of actual Pt and Ni loaded on the catalyst.

## 3 Results and Discussion

### 3.1 Structural Characterization of the Catalysts

**FIGURE 2:**
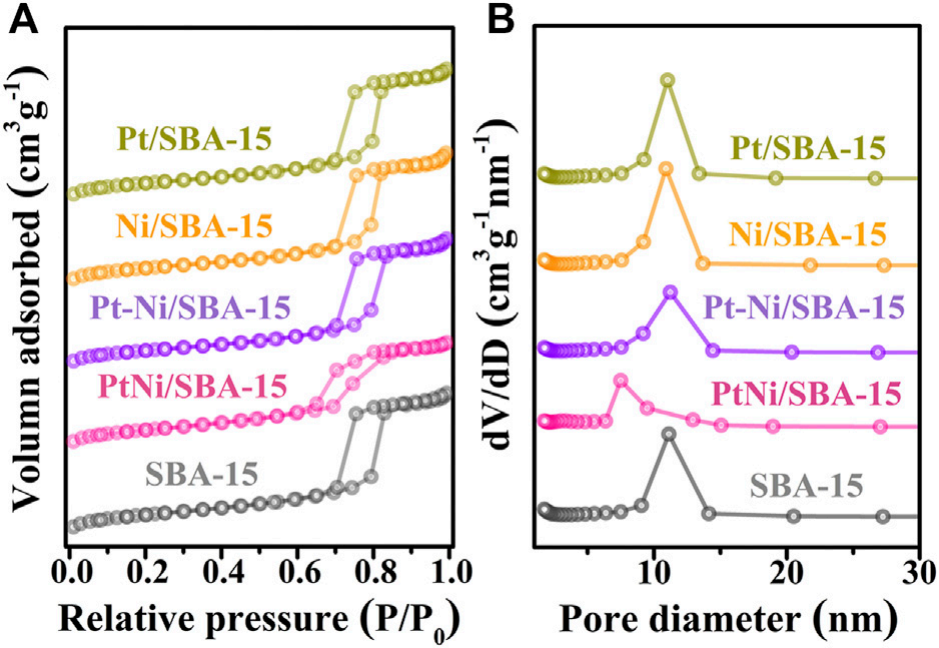
Nitrogen adsorption-desorption isotherms **(A)** and pore diameter distributions **(B)** of the synthesized catalysts and SBA-15.

The textural properties of pure silica SBA-15 and the synthesized catalysts were characterized by N_2_ adsorption-desorption measurement. The N_2_ adsorption-desorption isotherms and pore diameter distributions of all the samples were shown in Figure 2, and the corresponding textural properties of all the samples were summarized in Table 1. Nitrogen adsorption-desorption isotherms of these samples in Figure 2A were found to be type IV with a clear H1-type hysteresis loop. The steepness in the hysteresis loop revealed that these samples had ordered mesoporous structures (Goyal et al., 2016). Compared with pure silica SBA-15, the BET surface areas and pore volumes of all the synthesized catalysts had a decrease after metal introduction. The average pore diameter of PtNi/SBA-15 decreased obviously, due to the potential encapsulation of nanoalloys into the mesochannels(Chang et al., 2020). However, the average pore diameters of Pt/SBA-15, Ni/SBA-15, and Pt-Ni/SBA-15 slightly increased, which could be attributed to the blockage of the small micropores by nanoparticles (Zhou et al., 2020). These results confirmed the successful immobilization of metal on the SBA-15 support, and that PtNi alloys prepared by two-step anchored in the mesopores rather than filled the micropores as others catalysts prepared by incipient impregnation method.

**TABLE 1:** The physicochemical properties of the synthesized catalysts and SBA-15 support.

Catalyst	S_BET_ ^a^ (m^2^ g^−1^)	Pore volume (cm^3^ g^−1^)	D_pore_ ^a^ (nm)	D_TEM_ ^b^ (nm)	Metal loading^c^ (wt%)	Mole ratio Pt:Ni
PtNi/SBA-15	530.8	0.97	7.8	5.8	2.98	50.8:49.2
Pt-Ni/SBA-15	470.8	1.15	10.4	6.7	2.64	53.5:46.5
Pt/SBA-15	495.0	1.18	10.4	13.1	2.95	100:0
Ni/SBA-15	478.7	1.19	10.4	6.5	2.58	0:100
SBA-15	578.2	1.26	10.2	—	—	—

a S_BET_ and D_pore_ were the BET surface area and average pore diameter, obtained by nitrogen adsorption–desorption.

b Average diameter of nanoparticles, estimated from the images obtained by TEM.

c Metal loading of catalysts, confirmed by ICP-AES.

**FIGURE 3:**
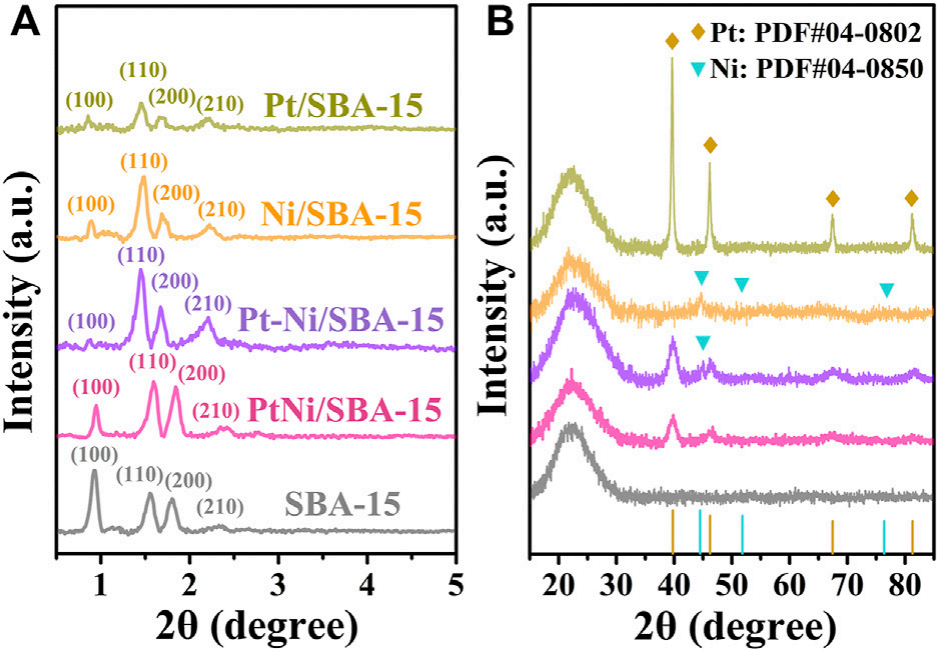
XRD patterns of different catalysts in the small-angle range **(A)** and wide-angle range **(B)**.

XRD analysis were employed to investigate the crystalline structures of the catalysts. The small-angle range of the XRD (SAXRD, 2θ = 0.5°–5°) patterns was shown in Figure 3A. The signals corresponding to the (100), (110), (200), and (210) planes of the 2D-hexagonal P6mm pore structure of SBA-15 support could be clearly observed (Jin et al., 2008). Although the signal intensity of (100) plane for these catalysts slightly decreased, the (110), (200) and (210) diffraction peaks were maintained, which revealed that the mesoporous structure of SBA-15 was preserved after metal loading. In addition, the SAXRD peaks of PtNi/SBA-15 shifted to a higher 2θ region, corresponding to a smaller d-spacing, which was attributed to the fact that a large number of nanoparticles loaded into the mesoporous pores leading to the structural shrinkage of the mesoporous silica framework (Goyal et al., 2016; Deng et al., 2019). In Figure 3B, wide-angle XRD (WAXRD, 2θ = 15°–85°) analysis was used to characterize the metal particles on the catalysts. Due to the amorphous silica structure of SBA-15, broad silica diffraction peaks at 22° were shown in all patterns. According to the literatures, the characteristic peaks at 2θ = 39.8°, 46.2°, 67.5°, and 81.3° were ascribed to face-centered cubic (fcc) Pt crystalline structure, while the diffraction peaks at 2θ = 44.5°, 51.8°, and 76.4° were attributed to fcc Ni crystalline structure (Mohan et al., 2012; Wu et al., 2012; Xu et al., 2014). The diffraction peaks of Pt/SBA-15 and Ni/SBA-15 were obviously related to the corresponding fcc Pt and fcc Ni structure, respectively. For PtNi/SBA-15 catalyst, the shifted diffraction peaks of Pt (observed at 2θ = 40.0°, 46.4°, 67.8°, and 81.4°) and the absence of peaks for Ni/NiO demonstrated the incorporation of Ni into Pt crystal and the generation of PtNi alloy. In comparison, weak diffraction peaks assigned to the (111) diffractions of fcc Ni crystal structure were observed for Pt-Ni/SBA-15, revealing a heteronuclear Pt-Ni bimetallic catalyst synthesized by traditional impregnation method.

**FIGURE 4:**
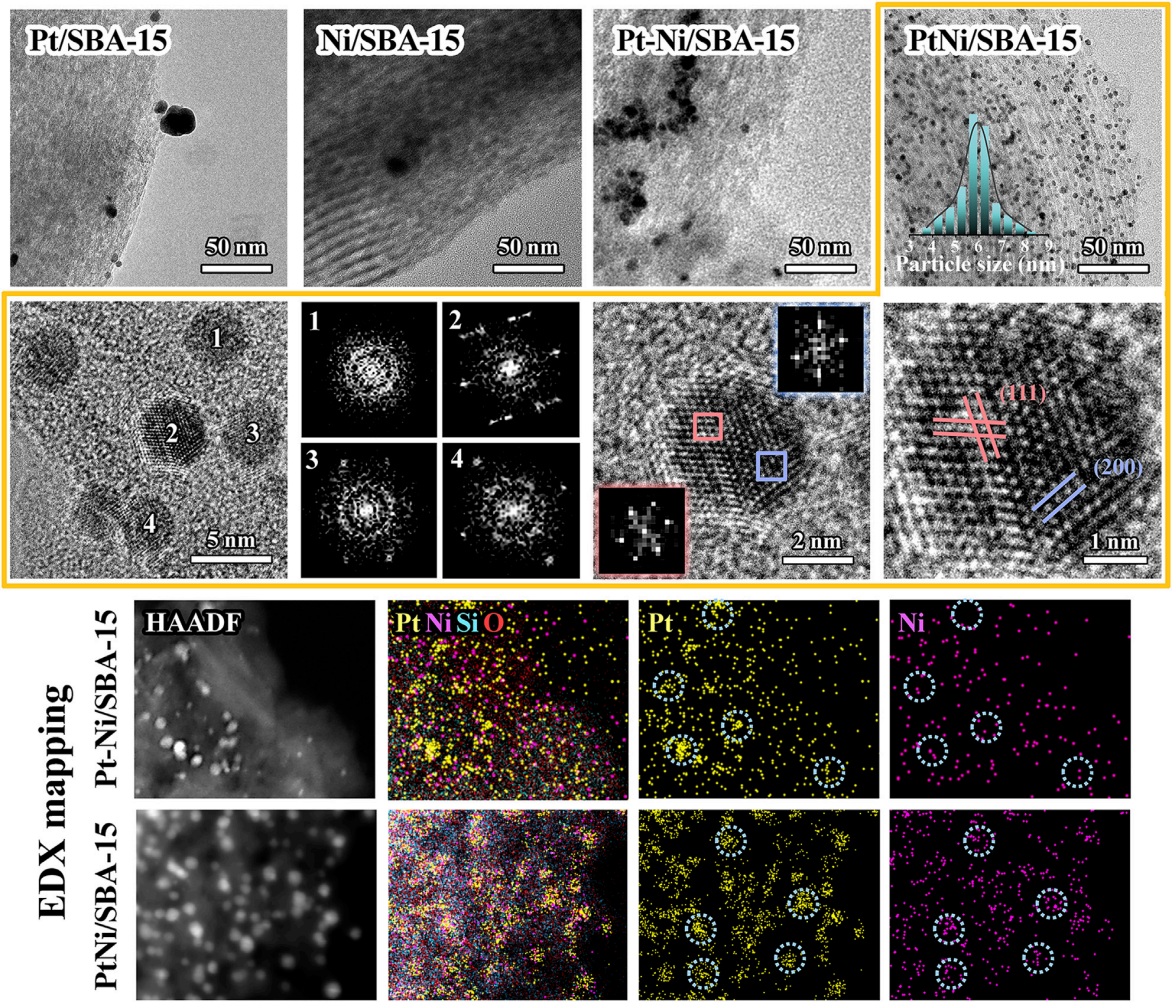
TEM images, HRTEM images, FFT diffraction diagrams, and EDX mappings of the synthesized catalysts.

The TEM images were presented in Figure 4 and Supplementary Figures S1, S2. The average diameter of the metal particles on the catalysts was listed in Table 1. Pt species on Pt/SBA-15 aggregated into large particles with low dispersion, and the average particle size was 13.1 nm. There were only a small amount of metal particles observed on Ni/SBA-15, indicating that part of Ni species could be introduced into the silica framework of SBA-15 (Tao et al., 2016). The composite Pt-Ni resulted in high dispersion of metal particles on Pt-Ni/SBA-15, and the average particle size (6.7 nm) was obviously smaller than pure Pt particles. However, the majority of metal particles of Pt-Ni/SBA-15 intensively grew on the edge of SBA-15, rather than being evenly distributed on the tunnel of SBA-15. These results indicated that the metal ions might migrate to the surface and agglomerate during the ordinary incipient impregnation process, which limited the metal dispersion and decreased the metal atom utilization to some extent (Chang et al., 2020). For PtNi/SBA-15 catalyst, small metal particles with 5.8 nm of average diameter were uniformly distributed on SBA-15, which could be attributed to the fact that PVP played an important role in the novel synthesis method of PtNi/SBA-15. In the hydrothermal synthesis process, PVP, as surfactant with the isolation function, contributed to form PtNi nanoalloys and control the PtNi alloys size, and structure (Xu et al., 2014). In the impregnation and calcination process, the use of PVP prevented the aggregation of metal particles and successfully fixed the metal particles in the SBA-15 pores channel, which inhibited their migration onto the surface (Chang et al., 2020). Furthermore, the smaller particle size of PtNi/SBA-15 would further increase the metal atom utilization and the active catalytic surface, which could minimize the metal usage and made the PtNi/SBA-15 catalyst more ecologically friendly while maintaining the efficient catalytic performance.

As shown in the representative high-resolution TEM (HRTEM) images (Figure 4; Supplementary Figure S2), numerous well-defined PtNi particles with visible lattice fringes could be identified on PtNi/SBA-15 catalyst. The fast Fourier transforms (FFT) shows the single-crystalline character, and the lattice spacing distances calculated by FFT were 0.23 and 0.19 nm, which were corresponding to the (111) and (200) planes for fcc PtNi alloy structure, respectively. Furthermore, the architectures of PtNi nanoalloys were further confirmed by high-angle annular darkfield scanning transmission electron microscopy (HAADF-STEM) images and corresponding EDX mappings. The most important difference between Pt-Ni/SBA-15 and PtNi/SBA-15 could be clearly observed in EDX mapping. For the part of metal particles on Pt-Ni/SBA-15, two types of metal signals could be identified but these signals usually showed in different areas respectively, which revealed the wide existence of Pt and Ni bulk particles. Nevertheless, signals of Pt and Ni were detected simultaneously for each metal particles on PtNi/SBA-15, confirming the existence of PtNi alloys on the catalyst.

**FIGURE 5:**
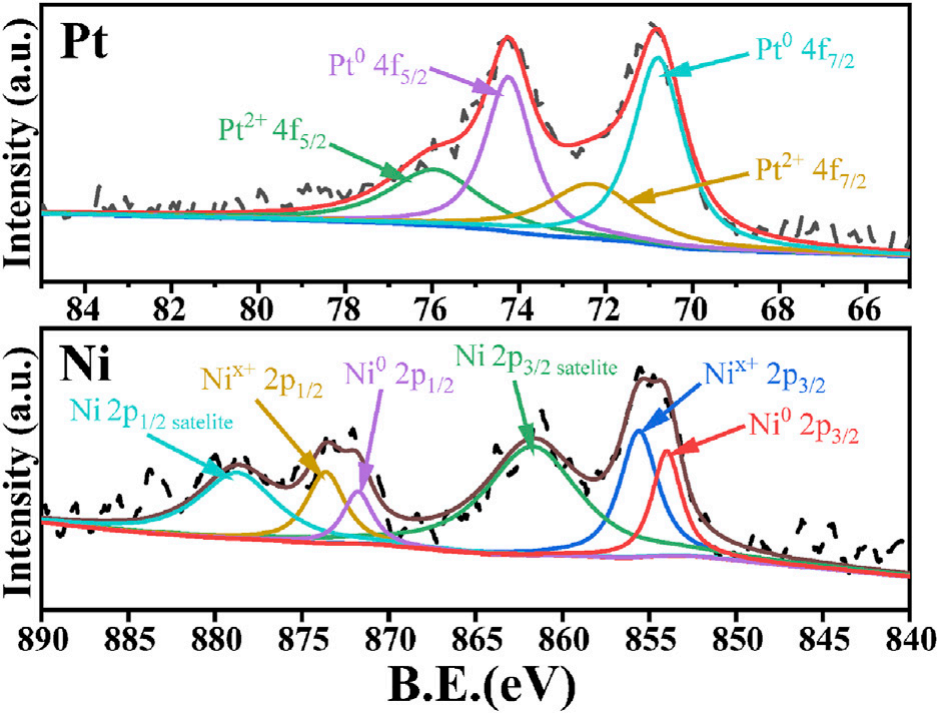
Pt 4f spectra and Ni 2p spectra of PtNi/SBA-15.

In order to further understand the interaction between Pt and Ni as well as the valence states of the metal species, XPS characterization of PtNi/SBA-15 was carried out and the spectra were presented in Figure 5. For PtNi/SBA-15, the Pt 4f XPS spectra exhibited two peaks assigned to Pt 4f_7/2_ (70.8 eV) and 4f_5/2_ (74.3 eV), the Ni 2p XPS spectra exhibited two peaks assigned to Ni 2p_3/2_ (854.6 eV) and 2p_1/2_ (872.5 eV). According to literature (Lan et al., 2019; Khalakhan et al., 2020), the binding energy of Pt was lower than that of pure Pt^0^ (4f_7/2_: 71.2 eV and 4f_5/2_: 74.5 eV), while the binding energy of Ni was higher than that of pure Ni^0^ (2p_3/2_: 852.7 eV and 2p_1/2_: 869.9 eV). The shifted binding energies of Pt and Ni could be attributed to the strong interaction between Pt and Ni. Thereafter, the charge transfer between the two metals led to the appearance of Pt^δ−^-Ni^δ+^ surface pairs on the PtNi alloys. The relative contents of Pt and Ni species with different valence states were summarized in Supplementary Tables S2, S3. The contents of Pt^0^ and Pt^2+^ were 70.8 and 29.8%, respectively, while the contents of Ni^0^ and Ni^x+^ in Ni species were 38.9 and 61.1%, respectively. However, nearly no XRD diffraction peaks could be identified for the oxide or hydroxide state of metal species in PtNi/SBA-15, which indicated that the oxide/hydroxide species were highly dispersed as amorphous state, rather than crystalline structure (Zhang and Wöllner, 2018).

### 3.2 Catalytic Hydrogenation of Furfural and 5-Hydroxymethylfurfural

**FIGURE 6:**
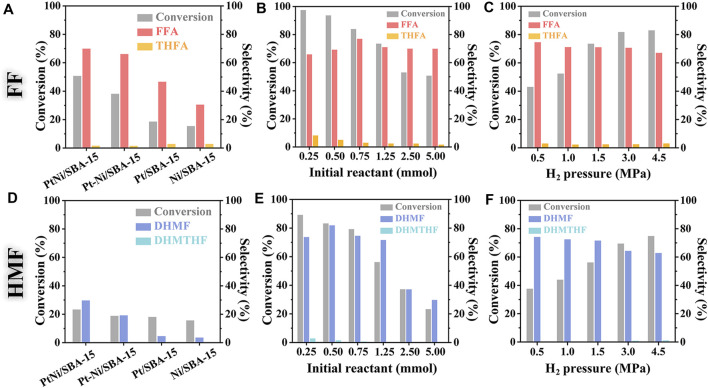
Catalytic hydrogenation of FF and HMF under different reaction conditions. Reaction conditions: 50 mg catalyst, 20 ml of H_2_O, 303 K, *t* = 2 h; **(A)** and **(D)**: 5 mmol FF/HMF, 1.5 MPa H_2_; **(B)** and **(E)**: 0.25∼5 mmol FF/HMF, 50 mg PtNi/SBA-15, 1.5 MPa H_2_; **(C)** and **(F)**: 1.25 mmol FF/HMF, 50 mg PtNi/SBA-15, 0.5∼4.5 MPa H_2_.

It was observed in Figure 6A that PtNi/SBA-15 exhibited the highest catalytic ability for the hydrogenation of FF over the other catalysts, and the conversion of FF reached 50.8% with 69.9% selectivity to FFA and only 1.7% selectivity to THFA. It should be mentioned that the hydrogenation process mainly occurred on the carbonyl, while the hydrogenation of C=C bond was hard to happen under these mild conditions. In comparison, the conversions of FF catalyzed by Pt-Ni/SBA-15, Pt/SBA-15 and Ni/SBA-15 were only 38.1, 18.6 and 15.4%, respectively, while the corresponding selectivities to FFA were 66.1, 46.6 and 30.5%. The conversion of FF and selectivity to FFA on PtNi/SBA-15 and Pt-Ni/SBA-15 were respectively higher than those on Pt/SBA-15and Ni/SBA-15. Although Pt-Ni/SBA-15 showed also acceptable selectivity, the conversion was much lower than PtNi/SBA-15. Similarly, the selective hydrogenation of HMF to DHMF was achieved by the catalysis of PtNi/SBA-15, whereas no 2,5-bis(hydroxymethyl)-tetrahydrofuran (DHMTHF) could be identified (Figure 6D). At molecular level, the catalytic reaction took place on the surface or interfaces of these catalysts, where reactants were adsorbed and subsequently converted to products that eventually desorbed from the catalyst surface. These SBA-15-supported catalysts had obvious selective C=O hydrogenation catalytic performance, but there were some other oligomers as side products detected by ESI-MS leading to poor molecular carbon balance. Therefore, the reaction conditions of FF/HMF hydrogenation catalyzed by PtNi/SBA-15 were optimized to improve the substrate conversion and product selectivity.

The effect of initial amount of reactant on the reaction was investigated (Figures 6B,E). Increasing the initial amount of reactant from 0.25 to 5.0 mmol (the molar ratio of reactant/metal increasing from 21:1 to 475:1), the conversion of FF declined from 97.4 to 50.8%, and the conversion of HMF attenuated from 89.3 to 23.4%, which suggested that high ratio of catalyst to substrate benefited to the conversion (Liu et al., 2018). However, the selectivity to FFA or DHMF increased firstly and then decreased with the initial amount of reactant, while the selectivity to THFA or DHMTHF monotonously decreased at the meantime. The product FFA/DHMF would be adsorbed by catalyst surface again under low substrate concentration, leading to over-hydrogenation to THFA/DHMTHF. These products and byproducts oligomers were detected by ESI-MS (Supplementary Figure S4). As reported in the literature, some oligomers such as furoin (Strassberger et al., 2010), 2-hydroxymethyl-5(5-furfuryl)furan, and difurfuryl ether (Kim et al., 2011) could be obtained during the reaction. The active sites were not enough to convert the excess substrates under high substrate concentration, so the conversion of FF/HMF to FFA/DHMF decreased and more oligomers have been obtained leading to poor molecular carbon balance.

H_2_ pressure was another critical parameter for hydrogenation process. The conversion of FF increased apparently from 43.1 to 83.0% with H_2_ pressure increasing in the range of 0.5∼4.5 MPa. The selectivity to FFA decreased from 74.7 to 67.1%, while the THFA selectivity showed a slight increase (Figure 6C). The hydrogenation of HMF exhibited similar trend of H_2_-controlled conversion and product selectivity (Figure 6F). These results suggested that higher H_2_ pressure promoted the hydrogenation of FF/HMF, especially triggering the hard-to-happen hydrogenation of C=C bond even at relatively low reaction temperature.

All the above results were summarized in Supplementary Tables S4, S5. Besides, reaction temperature and reaction time of the selective C=O hydrogenation were also investigated, and the results were shown in Supplementary Figures S8, S9. After optimization, 83.9% conversion of FF with 77.0% selectivity to FFA, and 83.3% conversion of HMF with 81.9% selectivity to DHMF were achieved over PtNi/SBA-15 with 1.5 MPa H_2_ pressure at 303 K for 2 h. Moreover, the TOF over PtNi/SBA-15 was calculated under the optimum conditions. The TOFs of FF and HMF conversion were 1,410 and 1,350 h^−1^, respectively, demonstrating that PtNi/SBA-15 exhibited satisfactory catalytic performance on the hydrogenation of the carbonyls in FF and HMF under mild conditions.

In recent years, efficient catalytic systems, which were similar to the PtNi/SBA-15 catalytic system, were widely studied for selective C=O hydrogenation of FF/HMF. For example, Fan et al. (2020) reported that 10 wt% Pt/C could catalyze 20% of FF conversion to FFA with 71.2% selectivity in tetrahydrofuran at 413 K under 2 MPa H_2_ pressure. Lima et al. (2017) reported that 44% selectivity to DHMF could be achieved over 10 wt% Pt/C at 373 K under 9 MPa H_2_ pressure. Srivastava et al. (2015) reported that 20 wt% Co-Cu/SBA-15 could catalyze FF hydrogenation to 80% yield FFA with 80% selectivity in isopropanol at 443 K under 2 MPa H_2_. Since plenty kinds of by-products were obtained *via* different reaction pathways under harsh conditions (Srivastava et al., 2015; Lima et al., 2017), the FFA/DHMF selectivities were often attenuated. Mild conditions could improve the selectivity to C=O hydrogenation and suppress the other side reactions (Chen et al., 2016; Chen et al., 2018). For example, Wu et al. (2021) reported that PtNi hollow nanoframes could catalyze the FF hydrogenation with 99% yield of FFA in isopropanol within 12 h at 373 K under 2 MPa H_2_. Xiao et al. (2021) reported that Pt_1_Sn_0.3_@HMSNs could yield 97% FFA in isopropanol within 5 h at 373 K under 1 MPa H_2_. However, these efficient catalytic systems need organic solvents as reaction medium, causing harm to environment, and limiting the hydrogenation application. It was reported by Wu et al. (2019) that FF hydrogenation catalyzed by Pt(3)Ni(1)/C could yield 80% FFA with 80% selectivity in aqueous phase within 12 h at 308 K under 2 MPa H_2_. Tan et al. (2019) reported that 92.7% yield of DHMF could be obtained by 12 wt% Pd/RGO catalyst after 6 h at 293 K under 1 MPa H_2_. Although the great catalytic performance have been achieved in aqueous phase under mild conditions, the catalytic efficiency and higher metal loading were not satisfactory, considering the usage of noble metal. Compared to reported catalytic systems, the advantages of the PtNi/SBA-15 catalytic system, such as nearly room temperature, lower H_2_ pressure, using water as green reaction medium without organic solvents, lower metal loading and great TOF, would promote the catalytic system to be employed in the future green industry.

### 3.3 Proposed Catalytic Mechanism

**FIGURE 7:**
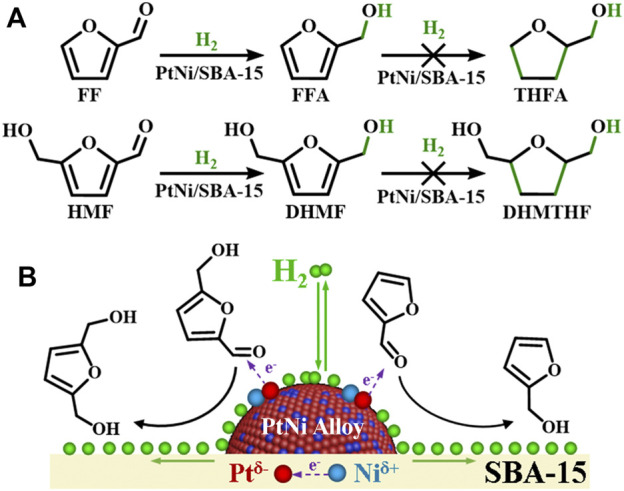
**(A)** Main reaction pathways for FF and HMF hydrogenation over the PtNi/SBA-15 catalyst. **(B)** Adsorption and activation of FF/HMF on the PtNi/SBA-15 catalyst.

In this catalytic system, gas hydrogen molecule was adsorbed and decomposed into activated hydrogen atoms on metal surfaces in the hydrogenation process, while reactants FF/HMF were adsorbed and subsequently converted to products that eventually desorbed from the catalyst surface. The pathway is governed by the geometric configuration and chemical bonding of substrate molecules on the surface, which could be controlled by the atomic and electronic structure of the surface or interface (Xie et al., 2020). PtNi/SBA-15 exhibited excellent catalytic performance in the selective carbonyl hydrogenation, restraining the further hydrogenation of the furan rings (Figure 7). The excellent catalytic performance of the C=O hydrogenation behavior could be ascribed to the charge transfer in PtNi alloy. Compared with monometallic catalysts (Pt/SBA-15 and Ni/SBA-15), bimetallic catalysts (PtNi/SBA-15 and Pt-Ni/SBA-15) have more efficient performance in the carbonyl hydrogenation due to the bimetallic synergy effect. Especially, PtNi/SBA-15 has more homogeneous bimetallic dispersion than Pt-Ni/SBA-15 as shown in Figure 4, leading to better catalytic activity. As confirmed by XPS analysis, Ni species acts as an electron donor in PtNi alloy, and thereby leads to the charge transfer between Pt and Ni. On one hand, the electron-rich Pt^δ−^ species in PtNi nanoalloys favors the adsorption and activation of C=O bond *via* donating electrons to the carbonyl groups. It has been found in reported literatures that the electron-rich species of bimetallic catalyst favors adsorption and activation of C=O bond in α,β-unsaturated aldehydes (Jiang et al., 2014; Tan et al., 2019; Wu et al., 2019). Due to strong Pt(5d)-CO(2π*) bonding interactions, the probability of C=O bond activation increases (Jiang et al., 2014). Meanwhile, the high electron density of Pt^δ−^ also weakens the strength of Pt-(C=C) interaction and decreases the activity of the C=C bond through an increase in the repulsive four-electron interaction (Jiang et al., 2014). On the other hand, the high electron density of the Pt in PtNi alloy is beneficial for the hydrogenation of FF/HMF due to the dissociation of H_2_ which needs the donation of d-denotation of Pt to hydrogen. The σ electrons of H_2_ could be accepted by metal Pt occupied d-orbitals, which donated d-electrons to the σ* antibonding orbital of H_2_, leading the H–H bond weakened and easily to be cleaved (Wu et al., 2021).

Moreover, hydrogen spillover is a phenomenon that the activated hydrogen atoms on the surface of active metal can migrate to the support (Merte et al., 2012; Karim et al., 2017; Yang et al., 2020). From the result of WO_3_ hydrogen spillover experiment as shown in Supplementary Figure S3, the colour change of yellow-to-black for PtNi/SBA-15 confirmed the existence of hydrogen spillover. Hydrogen spillover could further verify that PtNi/SBA-15 has excellent hydrogen activation performance and an amount of activated hydrogen atoms exists on catalyst under H_2_ atmosphere. The activated hydrogen atoms on SBA-15 support could also affect the selective hydrogenation of FF/HMF. It was found (Yang et al., 2020) that hydrogen spillover significantly promoted the C=O hydrogenation when the support could adsorb the C=O groups. SBA-15 has been reported to efficiently adsorb carbonyl groups (Huang et al., 2014). The carbonyl of FF/HMF adsorbed on SBA-15 could be hydrogenated to FFA/DHMF by the spillover activated hydrogen, further improving the hydrogenation selectivity of the carbonyls in FF/HMF.

These properties all co-contributed to the significant improvement of catalytic activity of the green and simple catalytic system over PtNi/SBA-15.

### 3.4 Stability of the PtNi/SBA-15 for the Furfural and HMF Hydrogenation

**FIGURE 8:**
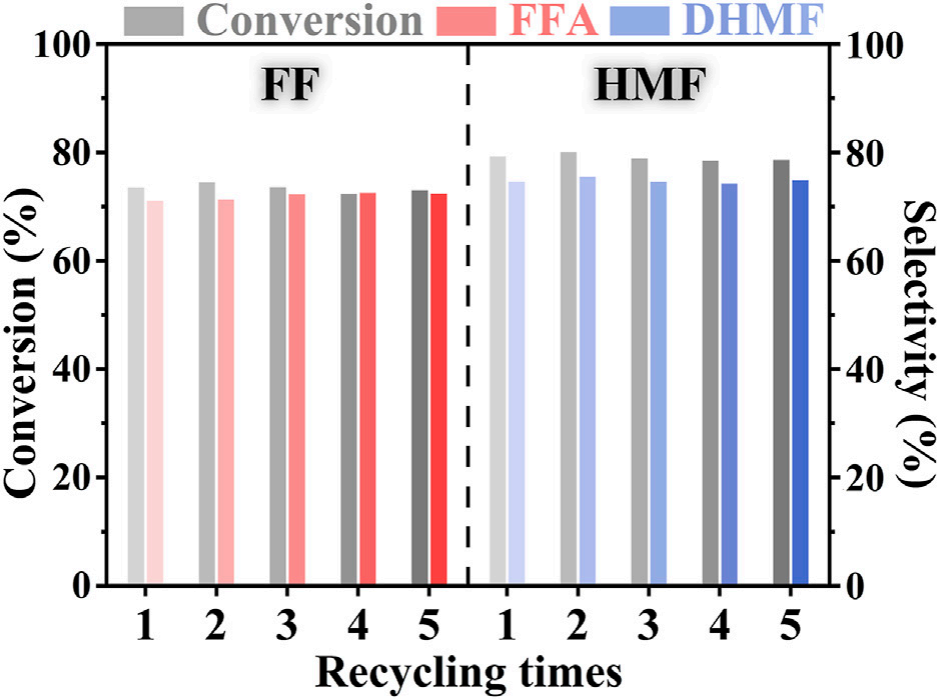
Reusability experiments of the PtNi/SBA-15 catalyst for the FF hydrogenation and the HMF hydrogenation. Substrate: 1.25 mmol FF or 0.75 mmol HMF. Reaction conditions: 303 K, 1.5 MPa H_2_, *t* = 2 h, 20 ml of H_2_O, 50 mg catalyst.

The recycling experiment of PtNi/SBA-15 catalyst was conducted to investigate the stability and reusability of the catalyst for the hydrogenation process under mild conditions. After each hydrogenation reaction, PtNi/SBA-15 catalyst could be easily separated from the reaction mixture by filtration. The recycled catalyst was only desiccated at 353 K without other treatment before another catalytic hydrogenation reaction. In Figure 8, it was noticeable that there was no remarkable decline for the conversions of FF/HMF and the selectivities to FFA/DHMF after five successive runs. These data of conversions and selectivities had some fluctuation within a narrow range due to the RPD in HPLC analysis. The yields of FFA/DHMF at the recycle experiments overall showed a slightly decreasing trend (53.2 ∼52.2% for FFA and 60.5 ∼58.8% for DHMF). The TEM of recycled PtNi/SBA-15 with main structure unchanged was shown in Supplementary Figure S10. These results indicated that PtNi/SBA-15 catalyst was stable and could be reused for the selective hydrogenation of carbonyls in FF and HMF under mild conditions. Furthermore, the easy separation, great stability and reusability of PtNi/SBA-15 minimized the waste of catalysts and the pollution to environment, further suggesting that the catalytic system over PtNi/SBA-15 is environmentally friendly and possible to expand application in the future.

## 4 Conclusion

In summary, SBA-15-supported bimetallic PtNi nanoalloys catalyst (PtNi/SBA-15) was successfully synthesized via a two-step method, and a green catalytic system for selective carbonyl hydrogenation of FF and HMF was explored under mild conditions. The prepared PtNi/SBA-15 catalyst maintains the ordered mesoporous structure with high surface area, on which PtNi nanoalloys with 5.8 nm average diameter were evenly dispersed. The electron-rich Pt^δ−^ species on PtNi/SBA-15 not only favors the adsorption and activation of the carbonyls in FF/HMF but also favors the activation of hydrogen molecule. Therefore, PtNi/SBA-15 could efficiently catalyze the hydrogenation of FF/HMF, obtaining FFA/DHMF with high selectivity in water under mild conditions (303 K, 1.5 MPa H_2_). The selectivities of 77.0% for FFA and 81.9% for DHMF were obtained over PtNi/SBA-15 from FF and HMF, respectively. Meanwhile, the TOFs over the PtNi/SBA-15 were as high as 1,410 h^−1^ for FF and 1,350 h^−1^ for HMF. In addition to its effectiveness, the PtNi/SBA-15 catalyst also displayed a high stability and reusability in FF and HMF hydrogenation at least five times without obvious deactivation. These findings open up the possibility for the synthesis of SBA-15-supported PtNi alloy catalyst with stable and good physicochemical properties, which can be adopted for the selective hydrogenation transformations of biomass-derived platform compounds. Also, this work towards the selective carbonyl hydrogenation of FF/HMF provides the possible protocol that could be used to design other green, stable, and efficient hydrogenation systems under mild conditions, with promising results that will be expanded to catalyze selective hydrogenation of biomass-derived materials in the future.

## Data Availability

The original contributions presented in the study are included in the article/Supplementary Material, further inquiries can be directed to the corresponding authors.

## References

[B1] Chacón-Huete F. Messina C. Chen F. Cuccia L. Ottenwaelder X. Forgione P. (2018). Solvent-free Mechanochemical Oxidation and Reduction of Biomass-Derived 5-hydroxymethyl Furfural. Green. Chem. 20, 5261–5265. 10.1039/c8gc02481b

[B2] Chang M. Liu X. Ning P. Zhang Q. Xia F. Wang H. (2020). Removal of Toluene over Bi-metallic Pt-Pd-SBA-15 Catalysts: Kinetic and Mechanistic Study. Microporous Mesoporous Mater. 302, 110111. 10.1016/j.micromeso.2020.110111

[B3] Chen C. Wang L. Zhu B. Zhou Z. El-Hout S. I. Yang J. (2021). 2,5-Furandicarboxylic Acid Production via Catalytic Oxidation of 5-hydroxymethylfurfural: Catalysts, Processes and Reaction Mechanism. J. Energ. Chem. 54, 528–554. 10.1016/j.jechem.2020.05.068

[B4] Chen J. Ge Y. Guo Y. Chen J. (2018). Selective Hydrogenation of Biomass-Derived 5-hydroxymethylfurfural Using Palladium Catalyst Supported on Mesoporous Graphitic Carbon Nitride. J. Energ. Chem. 27, 283–289. 10.1016/j.jechem.2017.04.017

[B5] Chen X. Zhang L. Zhang B. Guo X. Mu X. (2016). Highly Selective Hydrogenation of Furfural to Furfuryl Alcohol over Pt Nanoparticles Supported on G-C3n4 Nanosheets Catalysts in Water. Sci. Rep. 6, 28558. 10.1038/srep28558 27328834 PMC4916514

[B6] Deng T. Yan L. Li X. Fu Y. (2019). Continuous Hydrogenation of Ethyl Levulinate to 1,4‐Pentanediol over 2.8Cu‐3.5Fe/SBA‐15 Catalyst at Low Loading: The Effect of Fe Doping. ChemSusChem 12, 3837–3848. 10.1002/cssc.201901198 31218835

[B7] Fan Y. Li S. Wang Y. Zhuang C. Liu X. Zhu G. (2020). Tuning the Synthesis of Polymetallic-Doped ZIF Derived Materials for Efficient Hydrogenation of Furfural to Furfuryl Alcohol. Nanoscale 12, 18296–18304. 10.1039/d0nr04098c 32857827

[B8] Fang H. Yang J. Wen M. Wu Q. (2018). Nanoalloy Materials for Chemical Catalysis. Adv. Mater. 30, 1705698. 10.1002/adma.201705698 29450918

[B9] Fukuoka A. Dhepe P. L. (2006). Catalytic Conversion of Cellulose into Sugar Alcohols. Angew. Chem. Int. Ed. 45, 5161–5163. 10.1002/anie.200601921 16927334

[B10] Fulajtárova K. Soták T. Hronec M. Vávra I. Dobročka E. Omastová M. (2015). Aqueous Phase Hydrogenation of Furfural to Furfuryl Alcohol over Pd-Cu Catalysts. Appl. Catal. A: Gen. 502, 78–85. 10.1016/j.apcata.2015.05.031

[B11] Gandini A. (2010). Furans as Offspring of Sugars and Polysaccharides and Progenitors of a Family of Remarkable Polymers: a Review of Recent Progress. Polym. Chem. 1, 245–251. 10.1039/b9py00233b

[B12] Gandini A. (1990). Polymers and Oligomers Containing Furan Rings. Polym. Oligomers Containing Furan Rings 433, 195–208. 10.1021/bk-1990-0433.ch017

[B13] Goyal R. Sarkar B. Bag A. Siddiqui N. Dumbre D. Lucas N. (2016). Studies of Synergy between Metal-Support Interfaces and Selective Hydrogenation of HMF to DMF in Water. J. Catal. 340, 248–260. 10.1016/j.jcat.2016.05.012

[B14] Guo W. Liu H. Zhang S. Han H. Liu H. Jiang T. (2016). Efficient Hydrogenolysis of 5-hydroxymethylfurfural to 2,5-dimethylfuran over a Cobalt and Copper Bimetallic Catalyst on N-Graphene-Modified Al2O3. Green. Chem. 18, 6222–6228. 10.1039/c6gc02630c

[B15] Han X. Guo Y. Liu X. Xia Q. Wang Y. (2019). Catalytic Conversion of Lignocellulosic Biomass into Hydrocarbons: A Mini Review. Catal. Today 319, 2–13. 10.1016/j.cattod.2018.05.013

[B16] Hazlet S. E. Callison R. B. (1944). Crossed Cannizzaro Reactions-Benzaldehyde and Furfural1. J. Am. Chem. Soc. 66 **,** 1248–1250. 10.1021/Ja01236a007

[B17] Herrera C. Barrientos L. Rosenkranz A. Sepulveda C. García-Fierro J. L. Laguna-Bercero M. A. (2020). Tuning Amphiphilic Properties of Ni/Carbon Nanotubes Functionalized Catalysts and Their Effect as Emulsion Stabilizer for Biomass-Derived Furfural Upgrading. Fuel 276, 118032. 10.1016/j.fuel.2020.118032

[B18] Hui W. Zhou Y. Dong Y. Cao Z.-J. He F.-Q. Cai M.-Z. (2019). Efficient Hydrolysis of Hemicellulose to Furfural by Novel Superacid SO4H-Functionalized Ionic Liquids. Green. Energ. Environ. 4, 49–55. 10.1016/j.gee.2018.06.002

[B19] Jiang Z. Budarin V. L. Fan J. Remón J. Li T. Hu C. (2018). Sodium Chloride-Assisted Depolymerization of Xylo-Oligomers to Xylose. ACS Sustain. Chem. Eng. 6, 4098–4104. 10.1021/acssuschemeng.7b04463

[B20] Jiang Z. Zhao Y. Kong L. Liu Z. Zhu Y. Sun Y. (2014). Structure-Dependent Selective Hydrogenation of α,β-Unsaturated Aldehydes over Platinum Nanocrystals Decorated with Nickel. Chempluschem 79, 1258–1262. 10.1002/cplu.201402109

[B21] Jin Z. Wang X. Cui X. (2008). Synthesis and Morphological Investigation of Ordered SBA-15-type Mesoporous Silica with an Amphiphilic Triblock Copolymer Template under Various Conditions. Colloids Surf. A: Physicochemical Eng. Aspects 316, 27–36. 10.1016/j.colsurfa.2007.08.013

[B22] Karim W. Spreafico C. Kleibert A. Gobrecht J. Vandevondele J. Ekinci Y. (2017). Catalyst Support Effects on Hydrogen Spillover. Nature 541, 68–71. 10.1038/nature20782 28054605

[B23] Khalakhan I. Vega L. Vorokhta M. Skála T. Viñes F. Yakovlev Y. V. (2020). Irreversible Structural Dynamics on the Surface of Bimetallic PtNi alloy Catalyst under Alternating Oxidizing and Reducing Environments. Appl. Catal. B: Environ. 264, 118476. 10.1016/j.apcatb.2019.118476

[B24] Kijenski J. Winiarek P. (2000). Selective Hydrogenation of Alpha,beta-Unsaturated Aldehydes over Pt Catalysts Deposited on Monolayer Supports. Appl. Catal. A: Gen. 193, 1–4.

[B25] Kim T. Assary R. S. Marshall C. L. Gosztola D. J. Curtiss L. A. Stair P. C. (2011). Acid-Catalyzed Furfuryl Alcohol Polymerization: Characterizations of Molecular Structure and Thermodynamic Properties. ChemCatChem 3, 1451–1458. 10.1002/cctc.201100098

[B26] Lan J. Li C. Liu T. Yuan Q. (2019). One-step Synthesis of Porous PtNiCu Trimetallic Nanoalloy with Enhanced Electrocatalytic Performance toward Methanol Oxidation. J. Saudi Chem. Soc. 23, 43–51. 10.1016/j.jscs.2018.04.002

[B27] Lima S. Chadwick D. Hellgardt K. (2017). Towards Sustainable Hydrogenation of 5-(hydroxymethyl)furfural: a Two-Stage Continuous Process in Aqueous media over RANEY Catalysts. RSC Adv. 7, 31401–31407. 10.1039/c7ra03318d

[B28] Liu H. Huang Z. Kang H. Li X. Xia C. Chen J. (2018). Efficient Bimetallic NiCu-SiO2 Catalysts for Selective Hydrogenolysis of Xylitol to Ethylene Glycol and Propylene Glycol. Appl. Catal. B: Environ. 220, 251–263. 10.1016/j.apcatb.2017.08.022

[B29] Luo Y. Li Z. Li X. Liu X. Fan J. Clark J. H. (2019). The Production of Furfural Directly from Hemicellulose in Lignocellulosic Biomass: A Review. Catal. Today 319, 14–24. 10.1016/j.cattod.2018.06.042

[B30] Merte L. R. Peng G. Bechstein R. Rieboldt F. Farberow C. A. Grabow L. C. (2012). Water-Mediated Proton Hopping on an Iron Oxide Surface. Science 336, 889–893. 10.1126/science.1219468 22605771

[B31] Mika L. T. Cséfalvay E. Németh Á. (2018). Catalytic Conversion of Carbohydrates to Initial Platform Chemicals: Chemistry and Sustainability. Chem. Rev. 118, 505–613. 10.1021/acs.chemrev.7b00395 29155579

[B32] Mitra J. Zhou X. Rauchfuss T. (2015). Pd/C-catalyzed Reactions of HMF: Decarbonylation, Hydrogenation, and Hydrogenolysis. Green. Chem. 17, 307–313. 10.1039/c4gc01520g

[B33] Mohan V. Pramod C. V. Suresh M. Prasad Reddy K. H. Raju B. D. Rama Rao K. S. (2012). Advantage of Ni/SBA-15 Catalyst over Ni/MgO Catalyst in Terms of Catalyst Stability Due to Release of Water during Nitrobenzene Hydrogenation to Aniline. Catal. Commun. 18, 89–92. 10.1016/j.catcom.2011.11.030

[B34] Perez R. F. Fraga M. A. (2014). Hemicellulose-derived Chemicals: One-step Production of Furfuryl Alcohol from Xylose. Green. Chem. 16, 3942. 10.1039/c4gc00398e

[B35] Remón J. Santomauro F. Chuck C. J. Matharu A. S. Clark J. H. (2018). Production of Fermentable Species by Microwave-Assisted Hydrothermal Treatment of Biomass Carbohydrates: Reactivity and Fermentability Assessments. Green. Chem. 20, 4507–4520. 10.1039/c8gc02182a

[B36] Samikannu A. Konwar L. J. Rajendran K. Lee C. C. Shchukarev A. Virtanen P. (2020). Highly Dispersed NbOPO4/SBA-15 as a Versatile Acid Catalyst upon Production of Renewable Jet-Fuel from Bio-Based Furanics via Hydroxyalkylation-Alkylation (HAA) and Hydrodeoxygenation (HDO) Reactions. Appl. Catal. B: Environ. 272, 118987. 10.1016/J.Apcatb.2020.118987

[B37] Sharma R. V. Das U. Sammynaiken R. Dalai A. K. (2013). Liquid Phase Chemo-Selective Catalytic Hydrogenation of Furfural to Furfuryl Alcohol. Appl. Catal. A: Gen. 454, 127–136. 10.1016/j.apcata.2012.12.010

[B38] Shi S. Wu Y. Liu P. Zhang M. Zhang Z. Gao L. (2021). Efficient Conversion of Carbohydrates to 5-Hydroxymethylfurfural over Poly(4-Styrenesulfonic Acid) Catalyst. Catal. Lett. 10.1007/s10562-021-03693-7

[B39] Sitthisa S. Resasco D. E. (2011). Hydrodeoxygenation of Furfural over Supported Metal Catalysts: A Comparative Study of Cu, Pd and Ni. Catal. Lett. 141, 784–791. 10.1007/s10562-011-0581-7

[B40] Srivastava S. Mohanty P. Parikh J. K. Dalai A. K. Amritphale S. S. Khare A. K. (2015). Cr-free Co-Cu/SBA-15 Catalysts for Hydrogenation of Biomass-Derived α-, β-unsaturated Aldehyde to Alcohol. Chin. J. Catal. 36, 933–942. 10.1016/s1872-2067(15)60870-1

[B41] Strassberger Z. Mooijman M. Ruijter E. Alberts A. H. Maldonado A. G. Orru R. V. A. (2010). Finding Furfural Hydrogenation Catalysts via Predictive Modelling. Adv. Synth. Catal. 352, 2201–2210. 10.1002/adsc.201000308 23193388 PMC3501696

[B42] Sun Y. Fu T. Chen S. Wu Z. Guo Y. Pan D. (2019). A Novel Colorimetric Immunosensor Based on Platinum Colloid Nanoparticles Immobilized on PowerVision as Signal Probes and Fe 3 O 4 @ β ‐cyclodextrin as Capture Probes for Ractopamine Detection in Pork. J. Sci. Food Agric. 99, 2818–2825. 10.1002/jsfa.9492 30430588

[B43] Tan J. Cui J. Zhu Y. Cui X. Shi Y. Yan W. (2019). Complete Aqueous Hydrogenation of 5-Hydroxymethylfurfural at Room Temperature over Bimetallic RuPd/Graphene Catalyst. ACS Sustain. Chem. Eng. 7, 10670–10678. 10.1021/acssuschemeng.9b01327

[B44] Tao M. Meng X. Xin Z. Bian Z. Lv Y. Gu J. (2016). Synthesis and Characterization of Well Dispersed Nickel-Incorporated SBA-15 and its High Activity in Syngas Methanation Reaction. Appl. Catal. A: Gen. 516, 127–134. 10.1016/j.apcata.2016.02.019

[B45] Tian-Hui H. Zhao Y.-J. Yu-Juan Z. Zhao-Fu T. Xiao-Lan L. Qian L. (2014). Adsorption Properties of Ordered Mesoporous Silica for Butyraldehyde. Acta Physico-Chimica Sinica 30, 2307–2314. 10.3866/pku.whxb201410142

[B46] Wu J. Liu C. Zhu Y. Song X. Wen C. Zhang X. (2021). Understanding the Geometric and Electronic Factors of PtNi Bimetallic Surfaces for Efficient and Selective Catalytic Hydrogenation of Biomass-Derived Oxygenates. J. Energ. Chem. 60, 16–24. 10.1016/j.jechem.2020.12.011

[B47] Wu J. Zhang X. Chen Q. Chen L. Liu Q. Wang C. (2019). One-Pot Hydrogenation of Furfural into Tetrahydrofurfuryl Alcohol under Ambient Conditions over PtNi Alloy Catalyst. Energy Fuels 34, 2178–2184. 10.1021/acs.energyfuels.9b02811

[B48] Wu Y. Cai S. Wang D. He W. Li Y. (2012). Syntheses of Water-Soluble Octahedral, Truncated Octahedral, and Cubic Pt-Ni Nanocrystals and Their Structure-Activity Study in Model Hydrogenation Reactions. J. Am. Chem. Soc. 134, 8975–8981. 10.1021/ja302606d 22519877

[B49] Xiao T. Yan P. Li K. Yang C. Yu H. Wang J. (2021). Hollow Mesoporous Nanoreactors with Encaged PtSn Alloy Nanoparticles for Selective Hydrogenation of Furfural to Furfuryl Alcohol. Ind. Eng. Chem. Res. 60, 6078–6088. 10.1021/acs.iecr.1c00293

[B50] Xie C. Niu Z. Kim D. Li M. Yang P. (2020). Surface and Interface Control in Nanoparticle Catalysis. Chem. Rev. 120, 1184–1249. 10.1021/acs.chemrev.9b00220 31580651

[B51] Xu X. Zhang X. Sun H. Yang Y. Dai X. Gao J. (2014). Synthesis of Pt-Ni alloy Nanocrystals with High-index Facets and Enhanced Electrocatalytic Properties. Angew. Chem. Int. Ed. 53, a–n. 10.1002/anie.201406497 25195668

[B52] Yang Y. Liu H. Li S. Chen C. Wu T. Mei Q. (2019). Hydrogenolysis of 5-Hydroxymethylfurfural to 2,5-Dimethylfuran under Mild Conditions without Any Additive. ACS Sustain. Chem. Eng. 7, 5711–5716. 10.1021/acssuschemeng.8b04937

[B53] Yang Y. Wang Y. Li S. Shen X. Chen B. Liu H. (2020). Selective Hydrogenation of Aromatic Furfurals into Aliphatic Tetrahydrofurfural Derivatives. Green. Chem. 22, 4937–4942. 10.1039/d0gc01587c

[B54] Zhang G.-R. Wöllner S. (2018). Hollowed Structured PtNi Bifunctional Electrocatalyst with Record Low Total Overpotential for Oxygen Reduction and Oxygen Evolution Reactions. Appl. Catal. B: Environ. 222, 26–34. 10.1016/j.apcatb.2017.09.066

[B55] Zhao D. Feng J. Huo Q. Melosh N. Fredrickson G. H. Chmelka B. F. (1998). Triblock Copolymer Syntheses of Mesoporous Silica with Periodic 50 to 300 Angstrom Pores. Science 279, 548–552. 10.1126/science.279.5350.548 9438845

[B56] Zhou Y. Liu X. Yu P. Hu C. (2020). Temperature-tuned Selectivity to Alkanes or Alcohol from Ethyl Palmitate Deoxygenation over Zirconia-Supported Cobalt Catalyst. Fuel 278, 118295. 10.1016/j.fuel.2020.118295

[B57] Zhu Z. Ding D. Zhang Y. Zhang Y. (2020). Preparation of Ni, CoO-Supported Halloysite Nanotube Catalyst and its Application in the Hydrogenation of Furfural to Furfuryl Alcohol. Appl. Clay Sci. 196, 105761. 10.1016/j.clay.2020.105761

