# Supplementary material for: Selective Hydrogenation of the Carbonyls in Furfural and 5-Hydroxymethylfurfural Catalyzed by PtNi Alloy Supported on SBA-15 in Aqueous Solution Under Mild Conditions
Source: Front Chem. 2021 Sep 29;9:759512. doi: 10.3389/fchem.2021.759512 (PMC8511633; doi:10.3389/fchem.2021.759512)
Supplement: Supplementary file 1 [file Table1.DOCX]

Supplementary Material

Selective Hydrogenation of the Carbonyls in Furfural and 5-Hydroxymethylfurfural Catalyzed by PtNi Alloy Supported on SBA-15 in Aqueous Solution under Mild Conditions

**Ge Gao^1,2^,** **Zhicheng Jiang^1,2*^, Changwei Hu****^1,2,3*^**

^1^College of Biomass Science and Engineering, Sichuan University, Chengdu, P. R. China

^2^National Engineering Research Center of Clean Technology in Leather Industry, Sichuan University, Chengdu, P. R. China

^3^Key Laboratory of Green Chemistry and Technology, Ministry of Education, College of Chemistry, Sichuan University, Chengdu, P. R. China

| **Content** | **Subjects** | **Pages** |
| --- | --- | --- |
| Table S1 | The reported works for selective carbonyl hydrogenation of Furfural (FF) and 5-hydroxymethylfurfural (HMF). | 2 |
| Figure S1 | TEM images of Pt-Ni/SBA-15, Ni/SBA-15 and Pt/SBA-15. | 3 |
| Figure S2 | TEM and HRTEM images of PtNi/SBA-15. | 4 |
| Table S2.S3 | XPS Results of PtNi/SBA-15. | 5 |
| Table S4 | Results for the hydrogenation of FF over different catalysts. | 6 |
| Table S5 | Results for the hydrogenation of HMF over different catalysts. | 7 |
| Figure S3 | The photos of WO_3_ mixed with the synthesized catalysts after H_2_ treatment. | 8 |
| Figure S4 | The ESI-MS results of reacted solution. | 9 |
| Figure S5.S6 | The Formation Process of PtNi alloys. | 10 |
| Figure S7 | Thermogravimetric Analysis (TGA) of PtNi/SBA-15. | 11 |
| Figure S8.S9 | The Optimization of Reaction Conditions. | 12 |
| Table S6 | Results for the hydrogenation of FF/HMF over different catalysts and SBA-15 support. | 13 |
| Table S7 | Results for the C=O hydrogenation of furan compounds over PtNi/SBA-15 catalyst. | 14 |
| Figure S10 | The TEM images of PtNi/SBA-15 after hydrogenation reaction. | 15 |

**Table S1.** The reported works for selective carbonyl hydrogenation of Furfural (FF) and 5-hydroxymethylfurfural (HMF).

| **Substrate** | **Catalyst** | **Reaction Condition** | **Conv.(%)** | **Product** | **Sel. (%)** | **Reference** | |
| --- | --- | --- | --- | --- | --- | --- | --- |
| **FF** | **Cr-Cu** | **413 K, 720 Torr H_2_** | **60.0** | **FFA** | **80.0** | | **(**[**Rao et al., 1997**](#_ENREF_6)**)** |
| **FF** | **Cu:Cr (3:1)** | **443 K, 2 MPa H_2_, 3.5 h** | **75.0** | **FFA** | **60.0** | | **(**[**Sharma et al., 2013**](#_ENREF_7)**)** |
| **FF** | **Cu:Zn:Cr (3:2:1)** | **443 K, 2 MPa H_2_, 3.5 h** | **100** | **FFA** | **70.0** | | **(**[**Sharma et al., 2013**](#_ENREF_7)**)** |
| **FF** | **6.5% Ni-**  **(Hal-NA)** | **393 K, 2 MPa H_2_, 10 h** | **60.7** | **FFA** | **99.0** | | **(**[**Zhu et al., 2020**](#_ENREF_15)**)** |
| **FF** | **20% CuCo/Zn**  **@NPC-600** | **413 K, 2 MPa H_2_, 6 h** | **>99** | **FFA** | **100** | | **(**[**Fan et al., 2020**](#_ENREF_2)**)** |
| **FF** | **10% Pt/C** | **413 K, 2 MPa H_2_, 4 h** | **20.3** | **FFA** | **71.2** | | **(**[**Fan et al., 2020**](#_ENREF_2)**)** |
| **FF** | **5% Pd/C** | **413 K, 2 MPa H_2_, 4 h** | **65.6** | **FFA** | **50.4** | | **(**[**Fan et al., 2020**](#_ENREF_2)**)** |
| **FF** | **5% Pt/C** | **423 K, 2 MPa H_2_, 1.5 h** | **58.0** | **FFA** | **96.0** | | **(**[**Vaidya and Mahajani, 2003**](#_ENREF_10)**)** |
| **FF** | **20%CoCu/SBA-15** | **443 K, 2 MPa H_2_, 4 h** | **99.0** | **FFA** | **80.0** | | **(**[**Srivastava et al., 2015**](#_ENREF_8)**)** |
| **FF** | **5% Pt@CN** | **373 K, 1 MPa H_2_, 5 h** | **60.9** | **FFA** | **>99** | | **(**[**Chen et al., 2016**](#_ENREF_1)**)** |
| **FF** | **5% Pt@TECN** | **373 K, 1 MPa H_2_, 5 h** | **>99** | **FFA** | **>99** | | **(**[**Chen et al., 2016**](#_ENREF_1)**)** |
| **FF** | **PtNi Bimetal** | **373 K, 1 MPa H_2_, 1 h** | **26~99** | **FFA** | **22~99** | | **(**[**Wu et al., 2021**](#_ENREF_11)**)** |
| **FF** | **3% Pt 1% Ni/C** | **308 K, 2 MPa H_2_, 12 h** | **99** | **FFA** | **80** | | **(**[**Wu et al., 2019**](#_ENREF_12)**)** |
| **FF** | **3.4% Pt_1_Sn_0.3_@HMSNs** | **373 K, 1 MPa H_2_, 5 h** | **97.8** | **FFA** | **>99** | | **(**[**Xiao et al., 2021**](#_ENREF_13)**)** |
| **FF** | **3% PtNi/SBA-15** | **303 K, 1.5 MPa H_2_, 2 h** | **83.9** | **FFA** | **77.0** | | **This work** |
| **HMF** | **α-Al_2_O_3_** | **453 K, 2 MPa H_2_, 16 h** | **53.9** | **DHMF** | **42.3** | | **(**[**Guo et al., 2016**](#_ENREF_3)**)** |
| **HMF** | **5% Pd/C** | **393 K, 30 psi H_2_, 15 h** | **>95** | **DHMF** | **43.0** | | **(**[**Mitra et al., 2015**](#_ENREF_5)**)** |
| **HMF** | **RANEY® Ni** | **363 K, 90 bar h H_2_** | **100** | **DHMF** | **60.0** | | **(**[**Lima et al., 2017**](#_ENREF_4)**)** |
| **HMF** | **RANEY® Cu** | **363 K, 90 bar h H_2_** | **94.0** | **DHMF** | **79.0** | | **(**[**Lima et al., 2017**](#_ENREF_4)**)** |
| **HMF** | **5% Ru/C** | **363 K, 90 bar h H_2_** | **100** | **DHMF** | **27.0** | | **(**[**Lima et al., 2017**](#_ENREF_4)**)** |
| **HMF** | **10% Pt/C** | **363 K, 90 bar h H_2_** | **99.0** | **DHMF** | **44.0** | | **(**[**Lima et al., 2017**](#_ENREF_4)**)** |
| **HMF** | **1% Pt/SiO_2_** | **363 K, 90 bar h H_2_** | **69.0** | **DHMF** | **78.0** | | **(**[**Lima et al., 2017**](#_ENREF_4)**)** |
| **HMF** | **5% Pd/SiO_2_** | **363 K, 90 bar h H_2_** | **100** | **DHMF** | **17.0** | | **(**[**Lima et al., 2017**](#_ENREF_4)**)** |
| **HMF** | **5% Rh/Al_2_O_3_** | **363 K, 90 bar h H_2_** | **74.0** | **DHMF** | **81.0** | | **(**[**Lima et al., 2017**](#_ENREF_4)**)** |
| **HMF** | **12% Pd/RGO** | **293 K, 1 MPa H_2_, 6 h** | **77.4** | **DHMF** | **92.9** | | **(**[**Tan et al., 2019**](#_ENREF_9)**)** |
| **HMF** | **12% PdRu/RGO** | **293 K, 1 MPa H_2_, 6 h** | **99.9** | **DHMF** | **3.7** | | **(**[**Tan et al., 2019**](#_ENREF_9)**)** |
| **HMF** | **3% PtNi/SBA-15** | **303 K, 1.5 MPa H_2_, 2 h** | **83.3** | **DHMF** | **81.9** | | **This work** |

Conv. means the conversion of substrate. Sel. means the selectivity of the desired product. FFA: furfuryl alcohol, DHMF: 2,5-dihydroxymethylfuran.

**The TEM images of the Synthesized Catalysts**

**
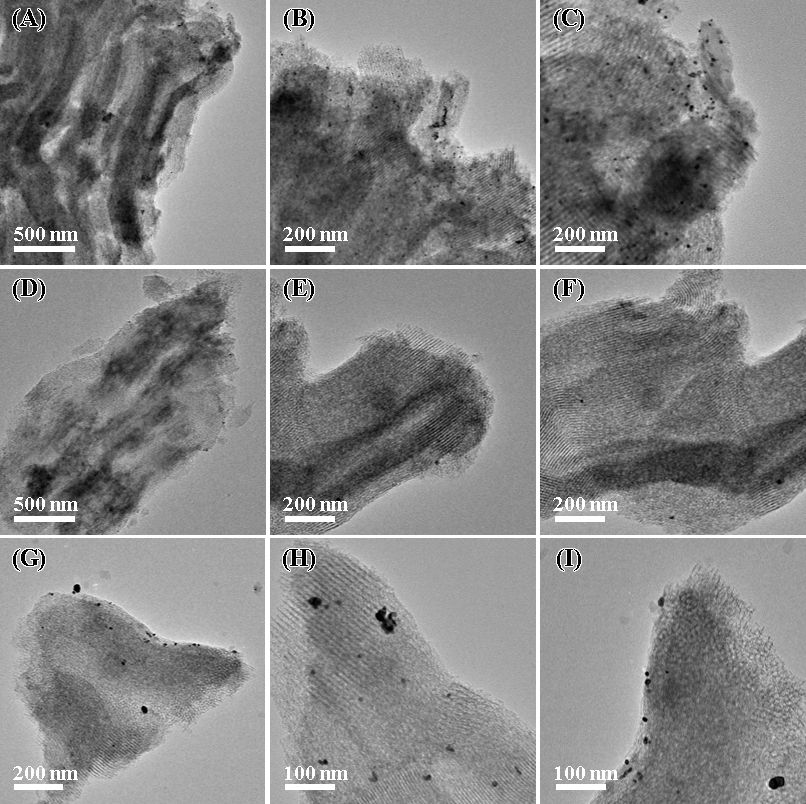
**

**Figure S1.** TEM images of Pt-Ni/SBA-15 (A-C), Ni/SBA-15 (D-F) and Pt/SBA-15 (G-I).

**
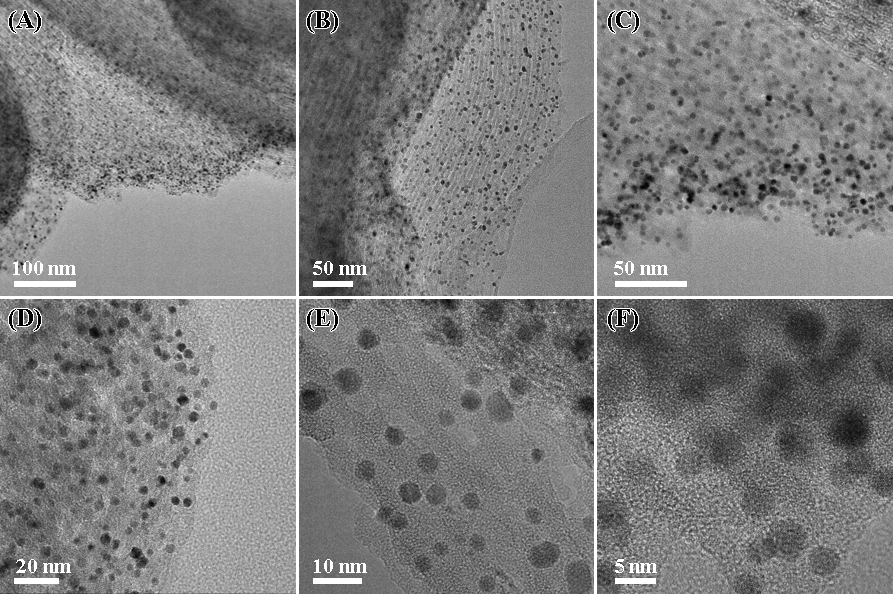
**

**Figure S2.** TEM (A-C) and HRTEM (D-F) images of PtNi/SBA-15.

**XPS results** **of PtNi/SBA-15**

**Table S2.** The binding energy and relative content for different species of Pt obtained from XPS results.

| **Species** | **B.E. (eV)^a^** | **Area** | **Content^b^** |
| --- | --- | --- | --- |
| **Pt^0^** | **70.8** | **809.4** | **70.8%** |
| **Pt^2+^** | **72.4** | **334.4** | **29.2%** |

^a^ Binding energy for Pt 4f_7/2_.

^b^ Determined from relative peak area of fitted XPS results.

**Table S3**. The binding energy and relative content for different species of Ni obtained from XPS results.

| **Species** | **B.E. (eV)^a^** | **Area** | **Content^b^** |
| --- | --- | --- | --- |
| **Ni^0^** | **854** | **332.2** | **38.9%** |
| **Ni^x+^** | **855.6** | **520.2** | **61.1%** |

^a^ Binding energy for Ni 2p_3/2_.

^b^ Determined from relative peak area of fitted XPS results.

**Catalytic hydrogenation results**

**Table S4.** Results for the hydrogenation of FF over different catalysts.^a^

| **Catalyst** | **Substrate (mmol)** | **Pressure (MPa)** | **Conv.**  **(%)** | **Selectivity (%)** | | | **FFA Yield (%)** | **TON^c^** |
| --- | --- | --- | --- | --- | --- | --- | --- | --- |
|  |  |  |  | **FFA** | **THFA** | **Others**^b^ |  |  |
| **PtNi/SBA-15** | **5.0** | **1.5** | **50.8** | **69.9** | **1.7** | **28.4** | **35.5** | **1364** |
| **Pt-Ni/SBA-15** | **5.0** | **1.5** | **38.1** | **66.1** | **1.5** | **32.4** | **25.2** | **1334** |
| **Pt/SBA-15** | **5.0** | **1.5** | **18.6** | **46.6** | **2.9** | **50.5** | **8.7** | **1956** |
| **Ni/SBA-15** | **5.0** | **1.5** | **15.4** | **30.5** | **2.9** | **66.6** | **4.7** | **224** |
| **PtNi/SBA-15** | **0.25** | **1.5** | **97.4** | **65.9** | **8.2** | **25.9** | **64.2** | **131** |
| **PtNi/SBA-15** | **0.50** | **1.5** | **93.7** | **69.3** | **5.1** | **25.6** | **64.9** | **251** |
| **PtNi/SBA-15** | **0.75** | **1.5** | **83.9** | **77.0** | **3.0** | **20.0** | **64.6** | **338** |
| **PtNi/SBA-15** | **1.25** | **1.5** | **73.6** | **71.1** | **2.6** | **26.3** | **52.4** | **494** |
| **PtNi/SBA-15** | **2.5** | **1.5** | **53.1** | **70.0** | **2.5** | **27.5** | **37.2** | **713** |
| **PtNi/SBA-15** | **1.25** | **0.5** | **43.1** | **74.7** | **2.7** | **22.6** | **32.2** | **289** |
| **PtNi/SBA-15** | **1.25** | **1.0** | **52.5** | **70.9** | **2.4** | **26.7** | **37.2** | **353** |
| **PtNi/SBA-15** | **1.25** | **3.0** | **81.8** | **70.7** | **2.5** | **26.8** | **57.8** | **549** |
| **PtNi/SBA-15** | **1.25** | **4.5** | **83.0** | **67.1** | **3.2** | **29.7** | **55.7** | **557** |
| **PtNi/SBA-15^d^** | **1.25** | **1.5** | **35.0** | **52.8** | **0.2** | **47.0** | **17.7** | **235** |

^a^ Reaction conditions: 50 mg catalyst, 20 mL of H_2_O, 303 K, H_2_ atmosphere, reaction time= 2 h. ^b^ Others contains a small amount of molecular product and some oligomers which undetected by HPLC. ^c^ TON means turnover number. ^d^ Reaction time: 10 min.

**Table S5.** Results for the hydrogenation of HMF over different catalysts.^a^

| **Catalyst** | **Substrate (mmol)** | **Pressure (MPa)** | **Conv.**  **(%)** | **Selectivity (%)** | | | **DHMF Yield (%)** | **TON­**^d^ |
| --- | --- | --- | --- | --- | --- | --- | --- | --- |
|  |  |  |  | **DHMF** | **DHMTHF** | **Others**^c^ |  |  |
| **PtNi/SBA-15** | **5.0** | **1.5** | **23.4** | **29.7** | **—^b^** | **70.3** | **6.9** | **629** |
| **Pt-Ni/SBA-15** | **5.0** | **1.5** | **18.9** | **19.3** | **—** | **80.7** | **3.6** | **587** |
| **Pt/SBA-15** | **5.0** | **1.5** | **18.1** | **4.6** | **—** | **95.4** | **0.8** | **1226** |
| **Ni/SBA-15** | **5.0** | **1.5** | **15.7** | **3.6** | **—** | **96.4** | **0.6** | **428** |
| **PtNi/SBA-15** | **0.25** | **1.5** | **89.3** | **73.8** | **2.9** | **23.3** | **65.9** | **120** |
| **PtNi/SBA-15** | **0.50** | **1.5** | **83.3** | **81.9** | **1.5** | **16.6** | **68.2** | **224** |
| **PtNi/SBA-15** | **0.75** | **1.5** | **79.3** | **74.6** | **0.7** | **24.7** | **59.2** | **320** |
| **PtNi/SBA-15** | **1.25** | **1.5** | **56.2** | **71.7** | **—** | **28.3** | **40.3** | **377** |
| **PtNi/SBA-15** | **2.5** | **1.5** | **37.2** | **37.2** | **—** | **62.8** | **13.8** | **500** |
| **PtNi/SBA-15** | **1.25** | **0.5** | **37.7** | **74.2** | **—** | **25.8** | **27.8** | **253** |
| **PtNi/SBA-15** | **1.25** | **1.0** | **44.0** | **72.5** | **—** | **27.5** | **31.9** | **295** |
| **PtNi/SBA-15** | **1.25** | **3.0** | **69.5** | **64.4** | **0.9** | **34.7** | **44.8** | **467** |
| **PtNi/SBA-15** | **1.25** | **4.5** | **74.9** | **62.9** | **1.2** | **35.9** | **47.1** | **503** |
| **PtNi/SBA-15^e^** | **1.25** | **1.5** | **33.5** | **47.5** | **—** | **52.5** | **15.9** | **225** |

^a^ Reaction conditions: 50 mg catalyst, 20 mL of H_2_O, reaction time= 2 h, 303 K, H_2_ atmosphere. ^b^ — refers to no product detected by HPLC. ^c^ Others contains a small amount of molecular product and some oligomers which undetected by HPLC. ^d^ TON means turnover number. ^e^ Reaction time: 10 min.

**Hydrogen spillover on the catalysts**


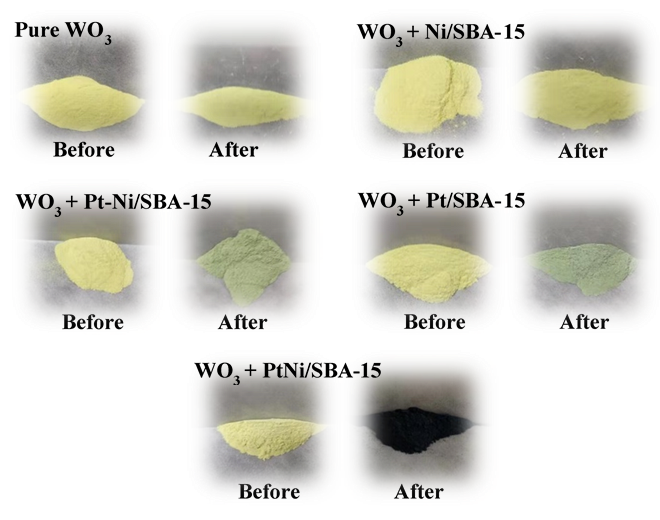
Hydrogen spillover is a common phenomenon that the activated hydrogen atoms on the surface of metal particles can further migrate to the support, which may make the support become the active centre as well.([Yang et al., 2020](#_ENREF_14)) Hydrogen spillover can be proved by using WO_3_ (yellow powder) to react with the migrated activated hydrogen to form H_x_WO_3_ (dark blue powder).([Yang et al., 2020](#_ENREF_14)) In our experiment, 10 mg of catalyst and 1 g WO_3_ were mixed and put into a transparent quartz tube. Then the mixture was H_2_-treated for 10 min at room temperature. In comparation, pure WO_3_ without catalyst was also tested under the same conditions. Pure WO_3_ and mixed Ni/SBA-15 samples remained the yellow colour after hydrogen treatment. However, the colour of the other samples turned from yellow to green. Especially, the treated mixture of WO_3_ and PtNi/SBA-15 turned to black, suggesting that more activated hydrogen atoms were obtained on PtNi/SBA-15. The results confirmed the existence of hydrogen spillover on the surface of Pt/SBA-15, Pt-Ni/SBA-15 and PtNi/SBA-15 as well as the better activated hydrogen performance of the PtNi alloys, and indicated that SBA-15 could dilute the activated hydrogen to the whole catalyst and make the catalysts more efficient in hydrogenation process.

**Figure S3.** The photos of WO_3_ mixed with the synthesized catalysts after H_2_ treatment.

**ESI-MS Qualitative Analysis**

The ESI-MS results of incompletely converted FF/HMF reaction solution were analysed in H_2_O system. ESI-MS results showed the remained reactants and their hydrogenation products. The oligomers were also detected, which confirmed that FF/HMF were partly converted to these oligomers rather than directly hydrogenated to FFA/DHMF.


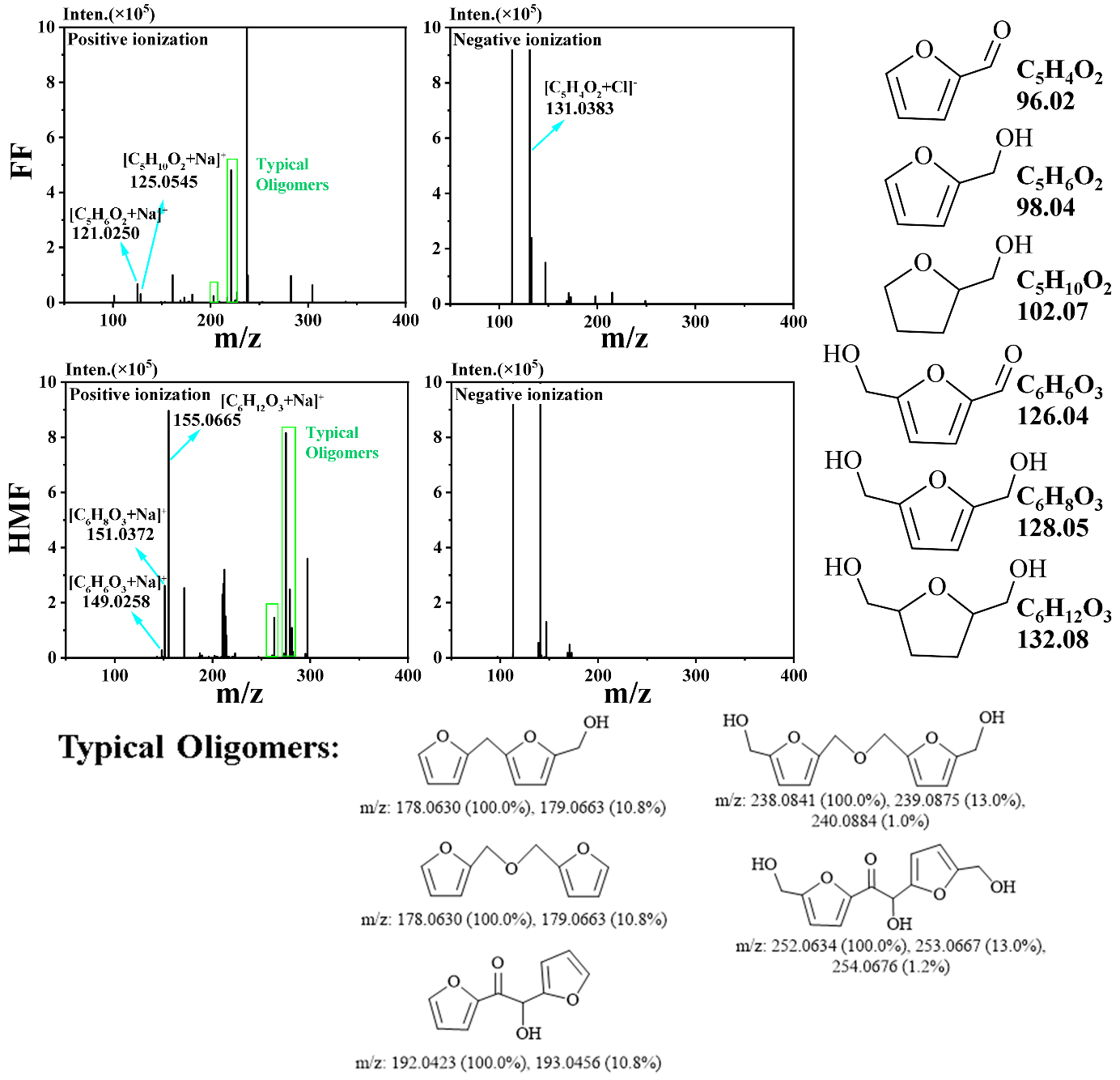


**Figure S4.** The ESI-MS results of reacted solution, measured by LCMS-IT-TOF (Shimadzu) using positive and negative ionizations. Reaction conditions: 0.5 mmol FF/HMF, 50 mg PtNi/SBA-15, 20 mL H_2_O, 1.5 MPa H_2_, 303 K and 2 h.

**
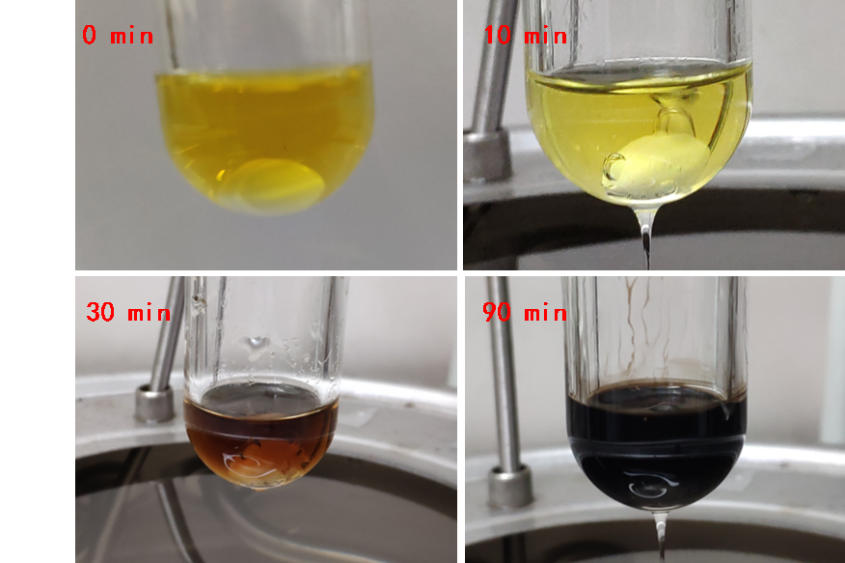
The Formation Process of PtNi alloys**

**Figure S5.** The photos of forming PtNi alloys by hydrothermal synthesis**.**


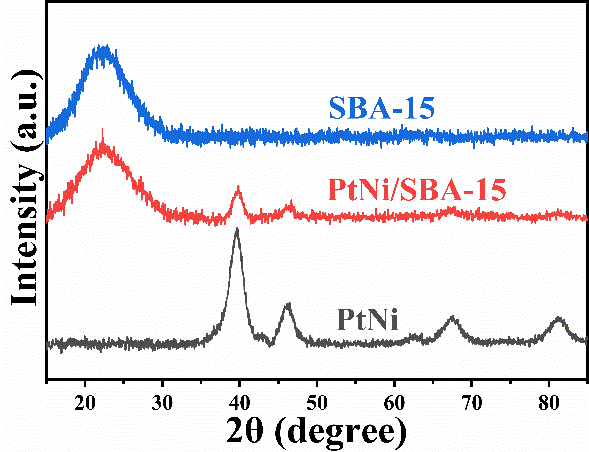

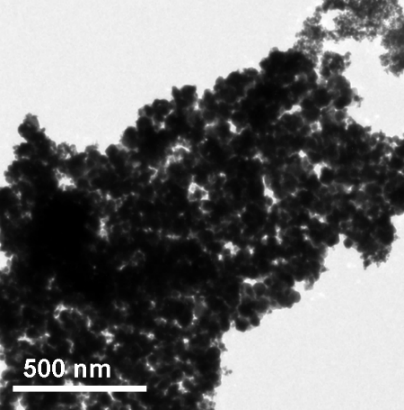

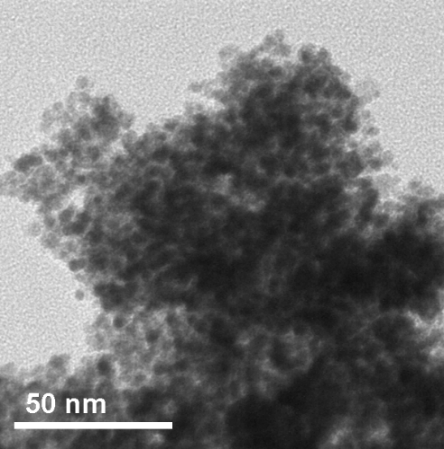

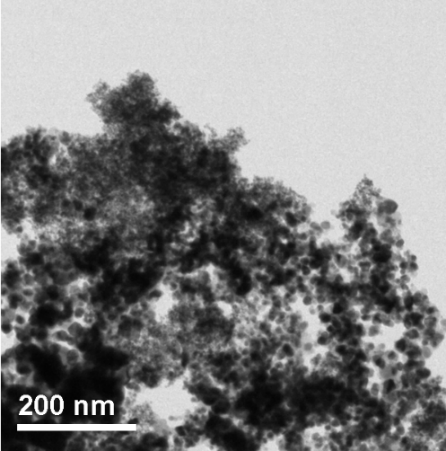


**Figure S6.** TEM and XRD of PtNi alloys without SBA-15 support after calcined.

**Thermogravimetric Analysis (TGA) of PtNi/SBA-15**

**Figure S7.** TGA and derivative thermogravimetric analysis (DTG) profiles of PtNi/SBA-15 uncalcined and PtNi/SBA-15.

**

The Optimization of Reaction Conditions**

**Figure S8.** The FF hydrogenation over PtNi/SBA-15 at different reaction temperature. Reaction conditions: 1.25 mmol FF, 50 mg PtNi/SBA-15, 20 mL of H_2_O, 1.5 MPa H_2_, reaction time = 2 h.

**

**

**Figure S9.** The FF hydrogenation over PtNi/SBA-15 at different reaction time. Reaction conditions: 1.25 mmol FF, 50 mg PtNi/SBA-15, 20 mL of H_2_O, 303 K, 1.5 MPa H_2_.

**The Effects of Solvents**

**Table S6.** Results for the hydrogenation of FF/HMF over different catalysts and SBA-15 support. ^a^

| **Catalyst** | **Solvent** | **Substrate (mmol)** | **Conv.^b^**  **(%)** | **Selectivity (%)** | | **FFA Yield (%)** |
| --- | --- | --- | --- | --- | --- | --- |
|  |  |  |  | **FFA** | **THFA** |  |
| **PtNi/SBA-15** | **H_2_O** | **1.25** | **73.6** | **71.1** | **2.6** | **52.4** |
| **PtNi/SBA-15** | **THF** | **1.25** | **26.2** | **29.7** | **3.1** | **7.8** |
| **PtNi/SBA-15** | **THF+H_2_O ^c^** | **1.25** | **42.9** | **30.2** | **1.6** | **12.9** |
|  |  |  |  | **Selectivity (%)** | | **DHMF Yield (%)** |
|  |  |  |  | **DHMF** | **DHMTHF** |  |
| **PtNi/SBA-15** | **H_2_O** | **0.75** | **79.3** | **74.6** | **0.7** | **59.2** |
| **PtNi/SBA-15** | **THF** | **0.75** | **27.3** | **42.9** | **— ^b^** | **11.7** |
| **PtNi/SBA-15** | **THF+H_2_O** | **0.75** | **60.1** | **65.1** | **—** | **39.1** |

^a^ Reaction conditions: 50 mg catalyst, 20 mL of solvent, 1.5 MPa H_2_ atmosphere, 303 K, 2h. ^b^ Conv. refers to conversion. — refers to no product detected by HPLC. ^c^ THF+H_2_O means THF: H_2_O (1:1, v/v) mixed solvent. THF means tetrahydrofuran.

From the results in Table S6, the experiments have obvious disparity that pure water as solvent is better than pure THF and THF mixed water for selective FF/HMF hydrogenation under the same conditions. In addition, water is environmentally friendly, energy-saving, and will not cause pollution. Therefore, we chose water as the solvent in this work.

**DFF (2,5-diformylfuran) Hydrogenation**

**Table S7.** Results for the C=O hydrogenation of furan compounds over PtNi/SBA-15 catalyst.^a^

| **Substrate** | **Conversion**  **(%)** | **Yield (%)** | | | | |
| --- | --- | --- | --- | --- | --- | --- |
|  |  | **FFA** | **THFA** | **DHMF** | **DHMTHF** | **HMF** |
| **FF** | **97.4** | **64.2** | **8.0** | **—** | **—** | **—** |
| **HMF** | **89.3** | **—^b^** | **—** | **65.9** | **2.6** | **10.7 (unreacted)** |
| **DFF^c^** | **99.9** | **—** | **—** | **58.4** | **—** | **5.9** |

^a^ Reaction conditions: 0.25 mmol substrate, 50 mg catalyst, 20 mL of H_2_O, 303 K, 1.5 MPa H_2_, reaction time= 2 h. ^b^ — refers to no product detected by HPLC. ^c^ DFF refers to 2,5-diformylfuran.

From the above results in Figure 5, the experiments have obvious disparity that FF was easier converted than HMF under the same conditions. It is speculated that this phenomenon was related to the substrate structure. To study how the substrate structure influenced these hydrogenation reactions, 2,5-diformylfuran (DFF) replaced FF or HMF as substrate for hydrogenation under the same conditions. As summarized in Table S7, these furan compounds could be hydrogenated easily over PtNi/SBA-15 under mild conditions. FFA and THFA were detected in the product of FF conversion, but only the C=O hydrogenation products could be detected in the HMF and DFF products, suggesting that more side-chain substituents could make the hydrogenation of furan ring harder. Moreover, the high ratio of carbonyl groups could promote the conversion of these furan compounds(DFF > FF > HMF). And the hydroxymethyl group of HMF, which made the HMF water-solubility greater than FF and DFF, sightly prejudiced to the HMF conversion to DHMF. These results further illustrated the PtNi/SBA-15 catalyst have efficient performance in the C=O selective hydrogenation.

**The TEM images of PtNi/SBA-15 after Hydrogenation Reaction**


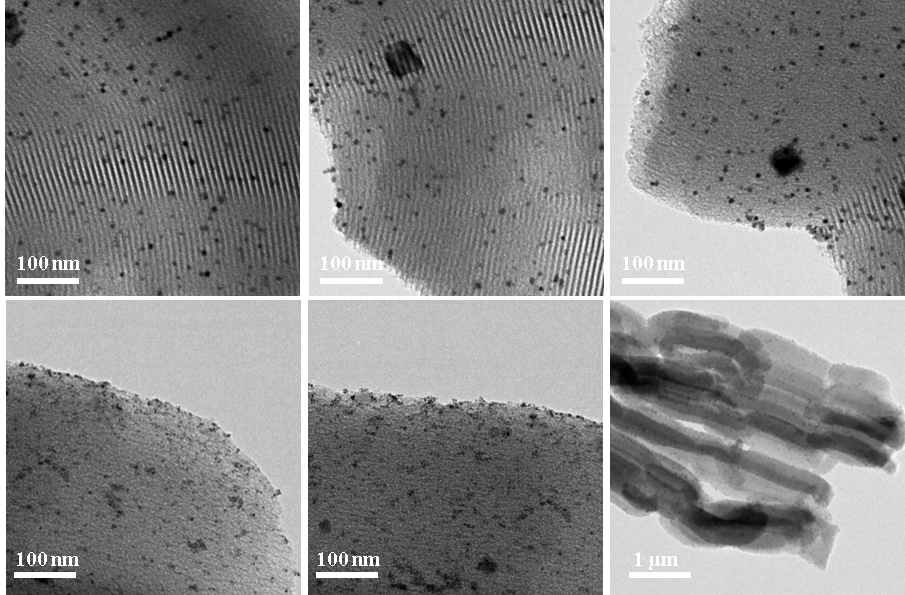


**Figure S10.** The TEM images of PtNi/SBA-15 after hydrogenation reaction (reaction time = 2 h).

**References**

Chen, X., Zhang, L., Zhang, B., Guo, X., and Mu, X. (2016). Highly selective hydrogenation of furfural to furfuryl alcohol over Pt nanoparticles supported on g-C_3_N_4_ nanosheets catalysts in water. *Sci Rep* 6**,** 28558. doi:10.1038/srep28558

Fan, Y., Li, S., Wang, Y., Zhuang, C., Liu, X., Zhu, G., and Zou, X. (2020). Tuning the synthesis of polymetallic-doped ZIF derived materials for efficient hydrogenation of furfural to furfuryl alcohol. *Nanoscale* 12**,** 18296-18304. doi:10.1039/d0nr04098c

Guo, W., Liu, H., Zhang, S., Han, H., Liu, H., Jiang, T., Han, B., and Wu, T. (2016). Efficient hydrogenolysis of 5-hydroxymethylfurfural to 2,5-dimethylfuran over a cobalt and copper bimetallic catalyst on N-graphene-modified Al_2_O_3_. *Green Chemistry* 18**,** 6222-6228. doi:10.1039/c6gc02630c

Lima, S., Chadwick, D., and Hellgardt, K. (2017). Towards sustainable hydrogenation of 5-(hydroxymethyl)furfural: a two-stage continuous process in aqueous media over RANEY® catalysts. *RSC Advances* 7**,** 31401-31407. doi:10.1039/c7ra03318d

Mitra, J., Zhou, X., and Rauchfuss, T. (2015). Pd/C-catalyzed reactions of HMF: decarbonylation, hydrogenation, and hydrogenolysis. *Green Chemistry* 17**,** 307-313. doi:10.1039/c4gc01520g

Rao, R., Dandekar, A., Baker, R.T.K., and Vannice, M.A. (1997). Properties of copper chromite catalysts in hydrogenation reactions. *Journal of Catalysis* 171**,** 406-419. doi:10.1006/jcat.1997.1832

Sharma, R.V., Das, U., Sammynaiken, R., and Dalai, A.K. (2013). Liquid phase chemo-selective catalytic hydrogenation of furfural to furfuryl alcohol. *Applied Catalysis A-General* 454**,** 127-136. doi:10.1016/j.apcata.2012.12.010

Srivastava, S., Mohanty, P., Parikh, J.K., Dalai, A.K., Amritphale, S.S., and Khare, A.K. (2015). Cr-free Co–Cu/SBA-15 catalysts for hydrogenation of biomass-derived α-, β-unsaturated aldehyde to alcohol. *Chinese Journal of Catalysis* 36**,** 933-942. doi:10.1016/s1872-2067(15)60870-1

Tan, J.J., Cuo, J.L., Zhu, Y.L., Cui, X.J., Shi, Y., Yan, W.J., and Zhao, Y.X. (2019). Complete Aqueous Hydrogenation of 5-Hydroxymethylfurfural at Room Temperature over Bimetallic RuPd/Graphene Catalyst. *Acs Sustainable Chemistry & Engineering* 7**,** 10670-10678. doi:10.1021/acssuschemeng.9b01327

Vaidya, P.D., and Mahajani, V.V. (2003). Kinetics of Liquid-Phase Hydrogenation of Furfuraldehyde to Furfuryl Alcohol over a Pt/C Catalyst. *Industrial & Engineering Chemistry Research* 42**,** 3881-3885. doi:10.1021/ie030055k

Wu, J., Liu, C., Zhu, Y., Song, X., Wen, C., Zhang, X., Wang, C., and Ma, L. (2021). Understanding the geometric and electronic factors of PtNi bimetallic surfaces for efficient and selective catalytic hydrogenation of biomass-derived oxygenates. *Journal of Energy Chemistry* 60**,** 16-24. doi:10.1016/j.jechem.2020.12.011

Wu, J., Zhang, X., Chen, Q., Chen, L., Liu, Q., Wang, C., and Ma, L. (2019). One-Pot Hydrogenation of Furfural into Tetrahydrofurfuryl Alcohol under Ambient Conditions over PtNi Alloy Catalyst. *Energy & Fuels* 34**,** 2178-2184. doi:10.1021/acs.energyfuels.9b02811

Xiao, T., Yan, P., Li, K., Yang, C., Yu, H., Wang, J., Yin, H., and Zhou, S. (2021). Hollow Mesoporous Nanoreactors with Encaged PtSn Alloy Nanoparticles for Selective Hydrogenation of Furfural to Furfuryl Alcohol. *Industrial & Engineering Chemistry Research* 60**,** 6078-6088. doi:10.1021/acs.iecr.1c00293

Yang, Y.D., Wang, Y.Y., Li, S.P., Shen, X.J., Chen, B.F., Liu, H.Z., and Han, B.X. (2020). Selective hydrogenation of aromatic furfurals into aliphatic tetrahydrofurfural derivatives. *Green Chemistry* 22**,** 4937-4942. doi:10.1039/d0gc01587c

Zhu, Z., Ding, D., Zhang, Y., and Zhang, Y. (2020). Preparation of Ni, CoO-supported halloysite nanotube catalyst and its application in the hydrogenation of furfural to furfuryl alcohol. *Applied Clay Science* 196**,** 105761. doi:10.1016/j.clay.2020.105761
